# The Effectiveness of Psychological Interventions for Rheumatoid Arthritis (RA): A Systematic Review and Meta-Analysis

**DOI:** 10.3390/life13030849

**Published:** 2023-03-21

**Authors:** Zsófia Nagy, Eszter Szigedi, Szabolcs Takács, Noémi Császár-Nagy

**Affiliations:** 1 Psychosomatic Outpatient Clinic, 1037 Budapest, Hungary; 2 General Psychology and Methodology, Faculty of Humanities, Károli Gáspár University of the Reformed Church, Bécsi Str. 324, 1037 Budapest, Hungary; 3 Department of Public Organization and Information Technology, Faculty of Public Governance, and International Studies, University of Public Service, Ludovika Square 2, 1083 Budapest, Hungary

**Keywords:** chronic pain, multimodal therapy, biopsychosocial treatment, CBT, mindfulness, relaxation, patient education, evidence-based

## Abstract

Rheumatoid arthritis (RA) is a long-term disorder that significantly impairs somatic, emotional, and psychological functioning. The objective of this review is to identify, appraise, and synthesize the effects of psychological interventions (e.g., cognitive behavioral therapy (CBT), emotional disclosure (ED), group therapy (GT), mindfulness (M), patient education (PE), and relaxation (R)) on biopsychosocial outcomes in the treatment of rheumatoid arthritis (RA). A systematic search of all relevant existing randomized clinical trials (RCTs) was conducted using the following online bibliographic databases: JSTOR, PubMed, PsycNET, and The Cochrane Library. Reference lists were searched for additional reports. The Cochrane Risk of Bias tool (RoB 2.0) was used to assess the risk of bias in the included studies. After the selection process, 57 articles were included and 392 were excluded. Three separate meta-analyses were conducted involving psychological interventions as the main variables, showing: (1) significant positive medium effect sizes for average values (Hedges-g = 0.399, Z = 0.399, *p* = 0.009); (2) significant positive large effect sizes for maximum values (Hedges-g = 0.856, Z = 4.223, *p* < 0.001); and (3) non-significant results for minimum values (Hedges-g = −0.047, Z = −0.335, *p* = 0.738). These results demonstrate that, when grouped, psychological interventions are, on average, moderately effective in treating RA. Overall, this review shows consistent, supportive evidence that psychological interventions can significantly contribute to the standard medical care of RA patients. However, more high-quality, large-sample RCTs still need to confirm these findings.

## 1. Introduction

### 1.1. Rheumatoid Arthritis (RA)

Rheumatoid arthritis (RA) is a medical condition that causes chronic joint inflammation and pain. The global prevalence of rheumatoid arthritis in 2010 was estimated at 0.24%, and interestingly, it was approximately twice as common in females (0.35%) than in males (0.13%) [1]. The condition can often damage various body systems, including the skin, eyes, lungs, heart, and blood vessels [2]. Since RA occurs when a person’s immune system mistakenly attacks the tissues in their body, it is categorized as an autoimmune disorder. Many people who have RA also experience other non-physical symptoms [3]. All these symptoms need to be addressed since, if left untreated, active RA can lead to a decreased quality of life, disability, and comorbidity [4]. Despite undergoing well-established pharmacological interventions, many patients with rheumatoid arthritis continue to experience various physical and psychological symptoms [1]. It is important to note that rheumatoid arthritis medications can also lead to side effects, especially when taken over long periods [1].

### 1.2. Psychology of RA

Patients suffering from this condition present various psychological symptoms (high levels of stress, negative mood, poor self-efficacy, maladaptive coping mechanisms, etc.) [3], which significantly affect their quality of life, their pain perceptions, and even their personality in the long term [5]. The psychological background of RA, including predisposing factors, premorbid traits, and consequent mental disorders, has been researched for a long time [6]. As for mental health disorders, approximately 20–40% of RA patients meet the criteria for a major depressive disorder, while 25–70% of RA patients present with an anxiety disorder [7]. Moreover, most patients exhibit both symptoms [7]. Research shows that these psychological factors have adverse outcomes in terms of both disease severity and activity [8] and are associated with increased pain [9,10], fatigue, and functional impairment [11].

### 1.3. Biopsychosocial Perspectives

With the increasing appreciation of the biopsychosocial approach to managing patients with chronic pain disease [12], more comprehensive treatment programs are starting to develop with less emphasis on biomedical aspects (i.e., interventional therapy and unimodal physical therapy) [13]. In its place, more and more interdisciplinary complex treatments focusing on functional restoration—those in which some form of social help or psychotherapy (such as vocational rehabilitation, relaxation training, occupational therapy) and somatic medicine are present at the same time [9]. Similarly, multiple psychometric tools are used in the assessment process and throughout the treatment to better assess the outcomes [14].

Since RA is categorically a chronic pain disease, its bio (inflammatory pain, fatigue), psycho (anxiety, depression), and social (low work capacity, less active lifestyle) natures should be well established. Therefore, it is essential for its treatment to be considered from the same perspective. The pharmacological treatment of RA has evolved substantially over the past few years, while research to develop complex treatments utilizing interdisciplinary teams remains limited [15]. These types of interventions are quite commonly researched in the treatment of chronic pain [16,17,18,19,20], but significantly fewer studies concentrate on a strictly RA population [21,22,23,24]. Moreover, currently, there are no available published descriptions of a comprehensive evidence-based treatment regime developed within an intensive interdisciplinary program to treat RA.

### 1.4. Psychological Interventions

Nowadays, the general aim of psychological treatments in RA is to attain a good quality of life, which includes, among other factors, an increase in self-efficacy, general mood, emotional and cognitive state, adaptive coping style, an active lifestyle, and work capability [22,25]. On the other hand, these psychological interventions are designed to decrease learned helplessness, catastrophizing, perceived pain, stress, interference with everyday activities, fatigue, functional and psychological disability, and psychological distress [25]. One of the main benefits of psychological interventions is their availability and applicability in various settings for the whole duration of the disease [9]. It is imperative to set clear treatment goals based on complex biopsychosocial diagnostics and to involve the patient in treatment decisions, which aims to improve both their adherence and compliance to achieve more successful treatment outcomes [26]. The psychosocial state of the patient influences their cognitive and emotional state, their overall mental health, their quality of life, and their perception/possible overestimation of disease activity [3].

According to the reviewed literature, [1,2,9,21,22,23,24] the main psychological interventions used in the treatment of RA and comorbid mental health disorders, and consequently the main variables of the study, are as follows:

(1) Patient education (PE). This intervention includes self-management training, coping skills training, modular behavioral education, and patient education [2,27]. In applied PE, there is a wide variety of tools depending on the individual goals of a program; however, most commonly, the importance of lifestyle modifications is discussed during these interventions. Education techniques mostly involve probing behavioral techniques and goal setting, along with participants committing to a specific behavior change for themselves [2]. PE trials in RA have shown that successful interventions significantly impact compliance and health outcomes [28,29,30]. PE interventions prove successful when they affect behavior change (not just provide information or advice) [28] or provide psychological education (not just general education programs [28,31].
(2) Stress management, relaxation, and basic psychotherapies (R). We treated relaxation and mindfulness-based interventions separately in our paper. The word relaxation originates from the Latin word release (relaxatum), thus narrowly the dissolution of a tense physical state; it means the relaxation of the muscles, which can be achieved with medicine and methods. Interventions in this group include relaxation techniques [32], counseling, supportive therapy, mindfulness [33], and self-regulation therapy [2]. Stress management programs mainly focus on stress-reducing effects, as these are psychological interventions aimed at modifying stress appraisal and decreasing the subjective perceptions of anxiety, which might alter autonomic arousal (e.g., decrease heart rate, breathe freely, and increase tonic vasodilation) and influence neuroendocrine activity [34]. Developing an adaptive coping method for the physiological response to a stressor could be particularly relevant in patients with immune-mediated diseases, such as RA [35].
(3) Cognitive behavioral therapy (CBT). CBT is a short-term method with a wide range of tools; it is evidence-based, well-established, and one of the most widely acknowledged psychological treatments available [36,37]. It helps patients improve their problem solving skills; achieve behavioral changes; and further reveal the relationship between beliefs, thoughts, and feelings, as well as the behaviors that stem from these factors [38]. Thus, it is also very appropriate for long-term personality development [38,39,40,41].
(4) Emotional disclosure (ED). In general, emotional disclosure consists of instructing patients to write and reflect individually and in private about their deepest thoughts and feelings regarding the most emotional event that they have experienced [42,43].
(5) Hypnotherapy (HY). Hypnotherapy is the psychotherapeutic application of clinical hypnosis, during which patients are in an altered state of consciousness, resulting in stress reduction and diversion of attention [44]. Therefore, it is suitable for triggering a lasting psycho-neuro-immunological response in addition to acute analgesia with targeted suggestions [44,45]. Consequently, it is a valuable method in the treatment of RA. Unfortunately, despite this, the use of hypnotherapy is less widespread and less researched than other psychotherapies, and the number of adequately conducted RCTs is also inadequate.
(6) Mindfulness (M): mindfulness interventions are psychological methods that originated from a Buddhist contemplative tradition. Mindfulness interventions can arouse inner concentration and improve self-regulation to alleviate patients’ psychological pressure, relieve pain response, and improve the quality of life [34]. Researchers have tried to apply mindfulness interventions to comprehensive treatment in various fields in recent years [16,34]. Mindfulness interventions have significant advantages as a relatively low-cost, non-invasive, and painless intervention without adverse reactions [46].
(7) Group therapy (GT): Group treatments can be linked to several psychotherapies in RA [35]. Their advantage is the power of the group to help each other (community of destiny), the group dynamics, and the care of several patients simultaneously [47]. Their disadvantage is that the patient receives less individual attention, so the strength of the therapist-client relationship may need to be stronger.

Based on the short introduction of the interventions, it is noticeable that there may be an overlap between these different psychological interventions; furthermore, psychological trends may also affect the application of specific methods (for example, the use of hypnotherapy in a cognitive approach), so, in any case, it may be worth carefully reviewing the detailed description of the intervention used. Further differences may arise from how the individual methods are implemented, such as in person or online; how much activity is required from the patient; how many sessions are required; the length of treatment; group or individual sessions; and the person holding the sessions.

### 1.5. Literature Review

Generally, most reviews focus on one specific psychological intervention (e.g., mindfulness [16,46]), self-management [14], or relaxation [17,44]). These interventions were shown to at least maintain, if not improve, the level of psychological well-being that would otherwise quickly deteriorate [1,2,21]. In other cases, they consider the efficacy of several methods but only on one variable, such as self-efficacy [48], fatigue [22], self-management [14], and depression or anxiety [24]. Moreover, many articles include a non-RA-specific population (e.g., chronic pain [17], other chronic health conditions [49], or headache [50]).

In addition, most studies do not include precise and detailed descriptions of interventions or the conduct of sessions. It is difficult to follow what previous education is, where the kind of intervention, how it was communicated to the persons, etc. was studied. Thus, the so-called placebo effects (patient expectations the role of conditioning is not always controlled). In addition, it is not always possible to follow the criteria according to which the intervention was considered interdisciplinary (because, in addition to medication, some physiotherapy was also administered, but elsewhere and without the specialists communicating, or the process took place in a team, etc.). Overall, no study would consider a sufficient number of factors (the patient’s side, the doctor’s influence, the relationship between the two, the precise administration of the methods, and the comparison of several psychological processes simultaneously). Such complex RCTs are notably lacking in the treatment of RA. Nevertheless, several RA-specific psychological interventions can be found in the literature and interdisciplinary treatment can be found, such as physiotherapy with medical therapy, nursing, or social care with medical treatment [51]. Still, we are determining if this happened in a team or simultaneously.

### 1.6. Objective

However, studies examining chronic-pain-specific adjuvant treatments in strictly RA populations are relatively rare. Therefore, to bridge this gap, we carried out this meta-analytic review of studies with psychological interventions in RA patients only. Overall, RA is a challenging condition as it is closely associated with socioeconomic disability [4] and high healthcare usage [47], as well as quickly deteriorating conditions in many areas of life [2]. Therefore, it can be essential to improve its treatments not only from a medical perspective but also from a complex biopsychosocial perspective as well. The following research questions related to the effectiveness of psychological treatments for RA were addressed as the main variables:

(1) Do psychological interventions (so-called psychotherapies), on average, affect the RA patient population?
(2) Could these psychological treatments be harmful in the worst case?
(3) What is the effect size of these interventions in the best possible cases?
(4) Are there any more effective, specific interventions? Are there any significant differences in the efficiency of the psychotherapeutic methods?

## 2. Materials and Methods

### 2.1. Protocol

**Figure 1:**
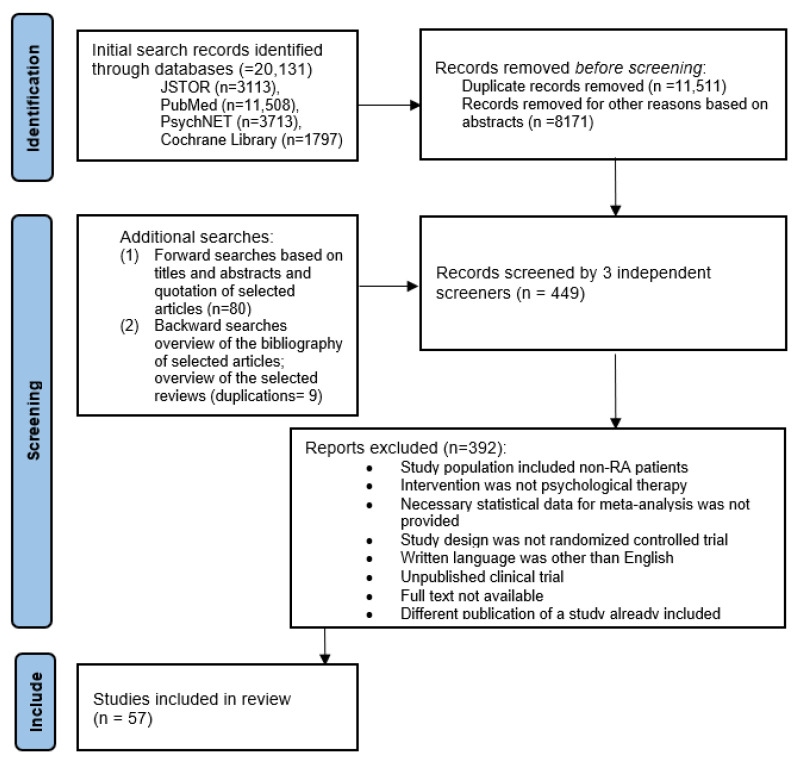
Flowchart of article screen and selection process (PRISMA).

The findings of this systematic review are reported based on the Preferred Reporting Items for Systematic Reviews and Meta-Analyses (PRISMA) reporting guideline [52]. Firstly, study eligibility and exclusion criteria are described. Secondly, all information sources and search strategies utilized throughout the search are specified, in addition to the detailed description of the process of article screening, coding, and the final selection of studies. Thirdly, the method of extracting and synthesizing data for the proposed meta-analyses based on the research questions is discussed. Lastly, statistical software is listed, and the main principles for combining raw data are explained. The PRISMA flowchart (Figure 1) shows the screening, selection process, and nine exclusion criteria.

### 2.2. Study Eligibility Criteria

This review was conducted based on the Cochrane Handbook for Systematic Reviews of Interventions [53]. First, both the inclusion and exclusion criteria were specified. The following eligibility criteria were applied based on the following PICOS components: (1) population: RA; (2) intervention: all types of psychological interventions; (3) comparison: control group; (4) outcome: quantitative results necessary for meta-analysis; (5) study: RCTs; (6) setting: all types of settings; (7) English full text. Conversely, studies were excluded if:

(1) the study population included non-RA patients;
(2) interventions were not psychological therapy;
(3) necessary statistical data for meta-analysis were not provided;
(4) the study design was not an RCT;
(5) the written language was anything other than English;
(6) the clinical trial was not published;
(7) the entire test was not available;
(8) a different publication of a study was already included.

### 2.3. Information Sources

Independent searches of the JSTOR, PubMed, PsychNET, and The Cochrane Library Central database were all conducted by two researchers. Citation management software, Zotero, and EndNote were used to import and eliminate studies and duplicates. The search was carried out on 29 March 2022.

### 2.4. Search Strategy

The following terms were used for conducting the online search of databases: [rheumatoid arthritis] AND [psychology OR psychotherapy OR mindfulness OR hypnosis OR relaxation OR imaginative therapy OR emotional disclosure OR self-management OR stress management OR cognitive and behavioral therapy]. No filters were applied during the search regarding the year of publication or any other variable.

### 2.5. Study Selection

In the first step, all the identified articles were scanned based on their titles, and abstracts were imported from the relevant database. Then, all those articles that did not meet the required inclusion and exclusion criteria for subsequent analysis were discarded. In cases where the previously defined criteria were met, the full texts were obtained.

Secondly, forward and backward searches were conducted based on the full-text articles obtained in the first round. Forward searches were carried out manually based on the titles and abstracts. Backward searching of bibliographies did not add any further articles. Additionally, unidentified RCTs that were analyzed in reviews found throughout the search were added to the screening process. Articles were screened in detail for eligibility. All full texts of the selected articles were reviewed by three independent researchers and analyzed based on the inclusion and exclusion criteria. The final number of studies reviewed item by item was 449, coded by three independent coders. Disagreements between the three independent coders were settled based on their professional consensus. Consequently, final decisions were made, resulting in 57 studies for the meta-analysis. The list of rejected articles can be found in Table S1.

### 2.6. Data Extraction and Data Synthesis

Four independent reviewers extracted and recorded the data in a pre-designed Excel datasheet. During the data collection, the four reviewers independently selected all studied variables, which included the following parameters for both baseline and post-intervention data:

- Mean (M);
- Standard deviation (SD);
- Standard error (SE);
- Confidence intervals (CI)s;
- Standard error measurement (SEM).

Furthermore, psychological interventions in the selected articles were grouped as follows:

- CBT (cognitive behavioral therapy);
- ED (emotional disclosure);
- GT (group therapy);
- M (mindfulness);
- PE (patient education);
- R (relaxation).

As for the eligible interventions, only those defined explicitly as psychological interventions could be included. In other words, they were acceptable if the methods used were proven to cause a change in mental state and psychosocial consequences. Lifestyle modification methods change living with the chronic conditions of RA and are therefore appropriate. Interventions consist of RA disease information, self-management, the management of medications, physical activity, coping strategies with the disease, emotional regulation, communication skills, personality development, cognitive symptom management, and improving social skills. We did not discriminate among interventions led by psychological or other healthcare professionals; however, the Table S2 summary table shows that psychologists or mental health professionals conducted most RCT clinical interventions.

### 2.7. Calculations and Analysis

For the statistical calculations, SPSS (version 27.0) [54] was used, and the meta-analyses were conducted using JASP (version 0.16.4) [55]. The following principles were applied during the statistical analysis to combine the raw data: case numbers were consistently replaced with the smallest case number in the study if the number of participants in any group was unclear, and higher SD values were considered. This resulted in a more rigorous analysis that improved the statistical adequacy of effect size calculations. A separate meta-analysis was conducted for each intervention group to determine the cumulative effect sizes (Hedges’ g). The homogeneity of included studies was examined using the I^2^ statistic.

## 3. Results

### 3.1. Study Characteristics

Throughout the search process, the three independent coders selected 57 studies from the initial search records for the meta-analysis. The article screening and study selection process is depicted in the PRISMA flowchart (Figure 1). Calculations of interrater reliability yielded a Cohen kappa of 0.624 (weighted, with a 95% confidence interval of 0.549–0.697), demonstrating an acceptable level of reliability for further analysis. All the selected studies were RCTs using RA populations. The total female percentage was 73.5, and the mean age was 49.65. Study characteristics are summarized in Table S2. The Table S2 summary table provides additional detailed information about the articles, the measuring instruments, and the results of the individual trials.

### 3.2. Risk of Bias

**Figure 2:**
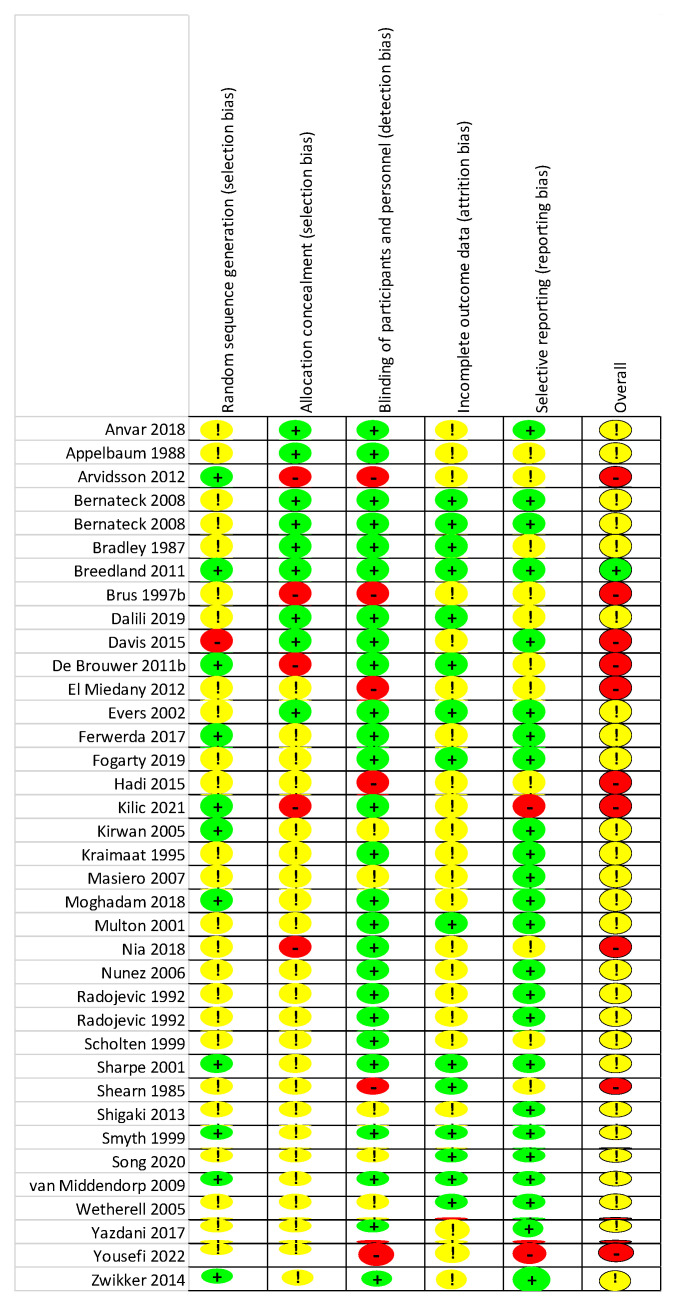
Risk of bias summary (per protocol): a review of the authors’ assessments of each risk of bias item for each included study. In these traffic light plots green indicates low risk of bias, while yellow indicates some concerns and red indicates high risk of bias.

**Figure 3:**
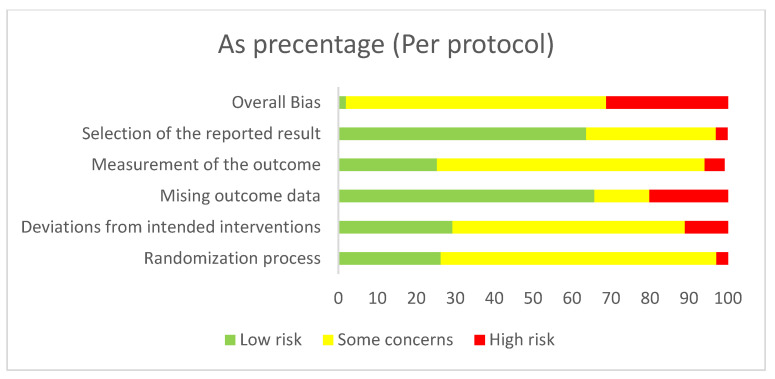
Risk of bias graph (per protocol): a review of authors’ judgements about each risk of bias item presented as percentages across all included studies.

**Figure 4:**
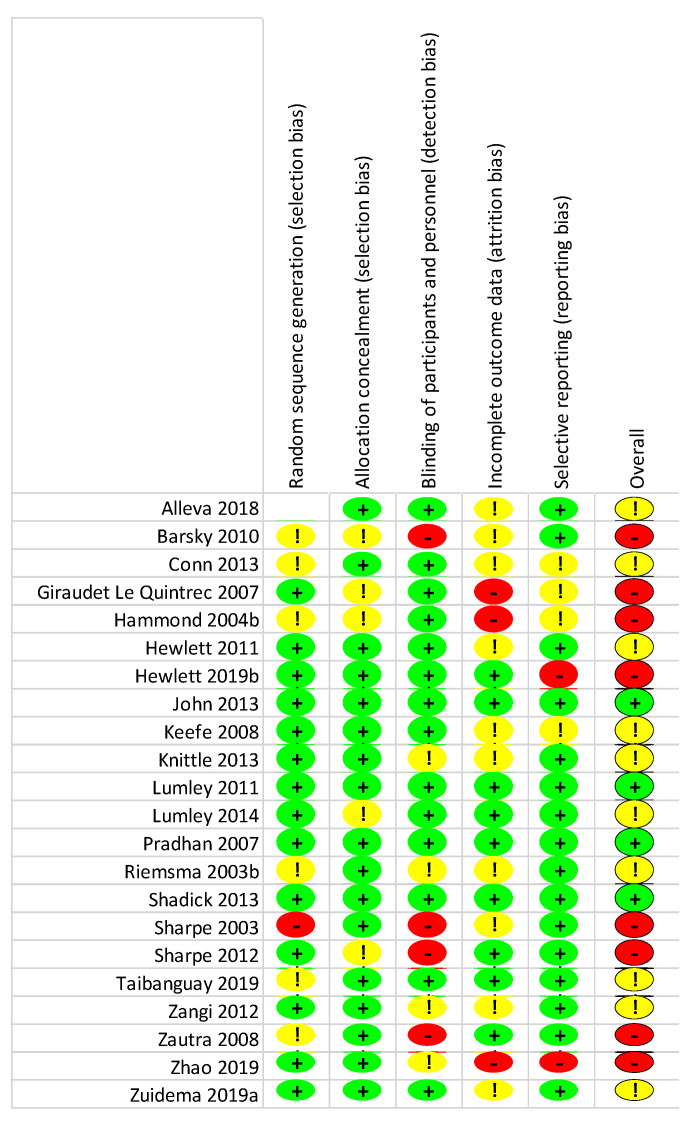
Risk of bias summary (intention-to-treat): a review of authors’ judgements about each risk of bias item for each included study. In these traffic light plots green indicates low risk of bias, while yellow indicates some concerns and red indicates high risk of bias.

**Figure 5:**
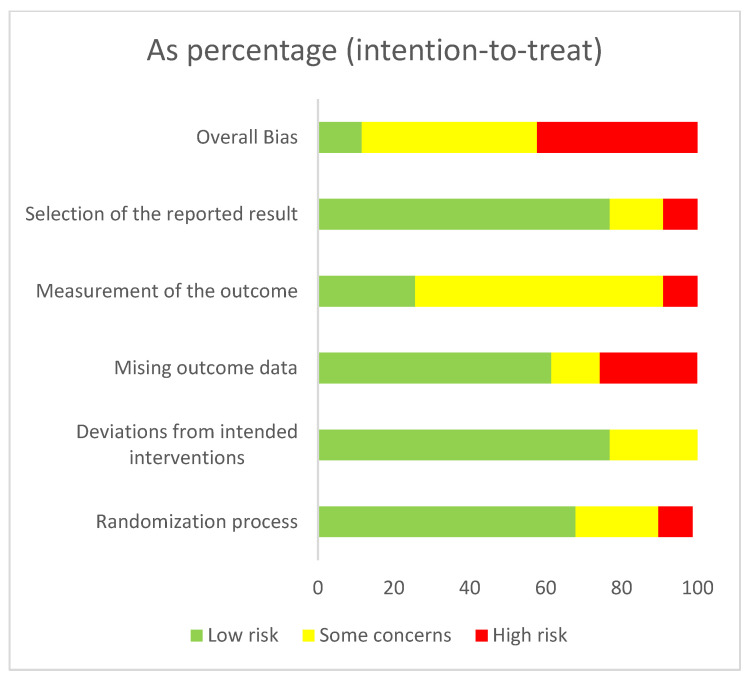
Risk of bias graph (intention-to-treat): a review of authors’ judgements about each risk of bias item presented as percentages across all included studies.

To assess the methodological quality of the included studies, the Cochrane Risk of Bias (RoB 2) tool was used [56]. This tool consists of five domains: sequence generation; allocation concealment; blinding of participants and personnel; incomplete outcome data; and selective reporting. Each criterion was evaluated separately for per-protocol and intention-to-treat designs based strictly on the published material and classified as either a having low risk of bias, some concerns, or a high risk of bias. Lastly, the domain bias ratings determined the overall risk of bias. There were some concerns about 51 studies (87%). Regarding the per-protocol studies, most concerns arose from randomized sequence generation (k = 26; 70%) or allocation concealment (k = 24; 65%). All in all, in ten studies (27%), there was a high risk of bias, while only one study (3%) had a low risk of bias. As for the intention-to-treat studies, incomplete outcome data (k = 13; 59%) and blinding (k = 8; 36%) were the signaling domains with the most concerns. Altogether, eight studies (36%), had a high risk of bias, while four had a low risk of bias (18%). Details of the risk of bias assessment are summarized in Figure 2, Figure 3, Figure 4 and Figure 5.

### 3.3. Effect of Intervention and Control Group

**Figure 6:**
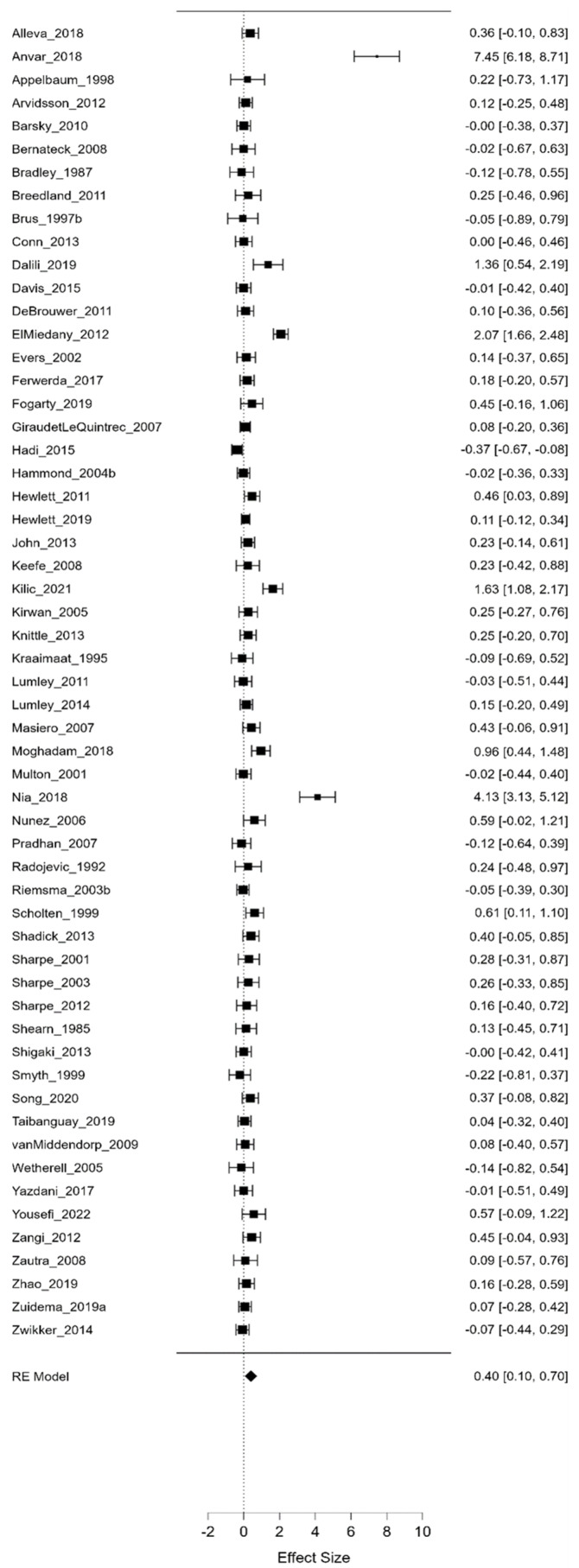
Average effect sizes (I^2^ = 95.809). In these figures square and diamond signs show effect sizes.

**Figure 7:**
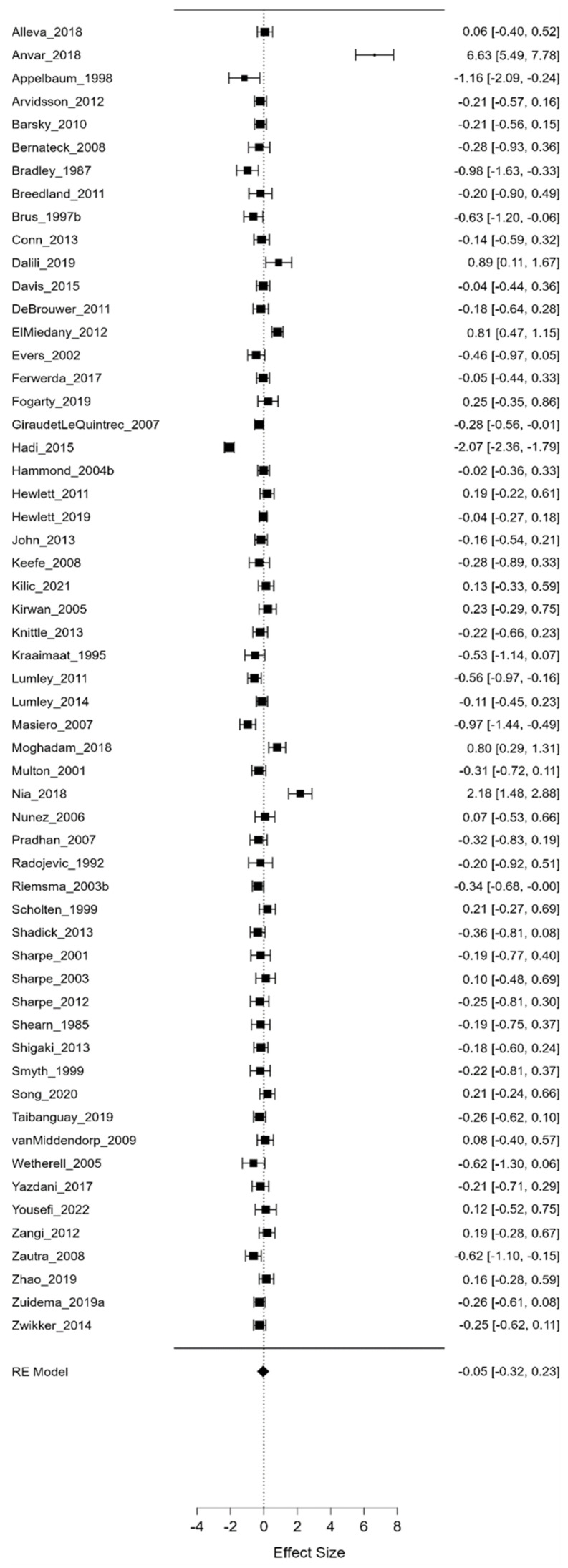
Minimum effect sizes (I^2^ = 95.289). In these figures square and diamond signs show effect sizes.

**Figure 8:**
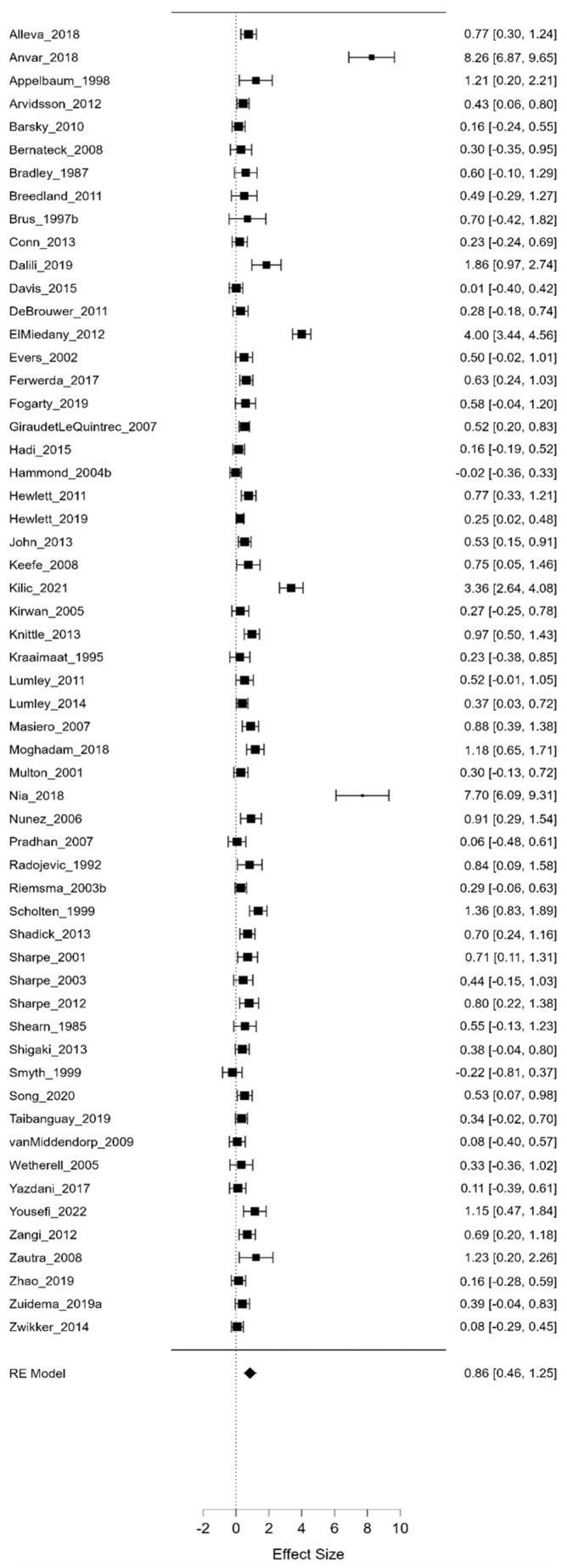
Maximum effect sizes (I^2^ = 97.383). In these figures square and diamond signs show effect sizes.

Three separate meta-analyses assessed psychological interventions’ average, maximum, and minimum efficacy. As illustrated in Figure 6, Figure 7 and Figure 8, we compared the effect sizes for all psychological interventions and looked at them separately (CBT, ET, GT, M, PE, and R).

As can be observed from the aggregated results, the average value of Hedges-g (Hedges-g = 0.399, Z = 0.399, *p* = 0.009) yielded a significant medium effect size, suggesting that, on average, psychological interventions are moderately effective. Moreover, the maximum value of Hedges-g (Hedges-g = 0.856, Z = 4.223, *p* < 0.001) yielded a large effect size, demonstrating that, in the best case, these psychological interventions can even be highly effective. On the other hand, the minimum value of Hedges-g (Hedges-g = −0.047, Z = −0.335, *p* = 0.738) and the effect sizes calculated for the different types of psychological interventions did not differ significantly from 0. Notably, this latter finding indicates that, while there is no evidence for the efficacy of psychological interventions under all circumstances, there are also no cases with significant adverse outcomes. Furthermore, comparing the average minimum and maximum values of Hedges-g presented in Figure 6, Figure 7 and Figure 8, it can be concluded that psychological interventions are the most effective. Table 1 shows the different effect sizes for all psychological interventions. 

## 4. Discussion

This systematic review and meta-analysis set out to analyze the available literature on psychological interventions used to treat RA. Numerous randomized control trials have been conducted in recent decades, primarily examining individual psychological interventions or a single psychological factor. Nevertheless, none analyzed the combination of a strictly RA population and all psychological interventions. The present review identified 57 randomized controlled trials with a strictly RA population. In the selected articles, a few mixed psychological interventions also appeared [21,23], but most studies focused on one method, such as CBT [40,41] or emotional disclosure [42]. Our findings align with prior studies describing psychological interventions’ positive impact on RA [2,23,43].

RA is a complex chronic disease that affects the entire functioning personality. The personality complex prone to rheumatism has not been confirmed [57]. By confirming that psychological interventions improve the condition of patients to such a large extent, we can assume that psychological factors are also behind and prone to the development of the disease. However, anxiety, depression, self-esteem disorders, difficulties in daily life, helplessness, dependence on others, decreased self-esteem, changes in social activities, and the possible transformation of relationships have also been shown to be significantly debilitating [21,34]; high pain perception (VAS) and pain catastrophizing are the most consistent predictors of poor somatic prognosis [6]. Minor distress in the patients’ lives already affects inflammatory processes. Natural life events themselves (e.g., change of residence) are also provocative, not to mention serious life events (e.g., death of a relative), which can prove to create an unfavorable clinical condition in patients who have difficulty processing their emotions [5,6,57]. It is not easy to separate causes and effects. The consequences of the disease are limited mobility, difficulties in everyday life, helplessness, dependence on others, a decrease in self-esteem, changes in social activities, and possible transformation of family relationships, so the condition causes distress and, thus, a self-stimulating process can begin. Optimism, tolerance for illness, positive faith in the future, life goals, and little self-blame are all desirable coping methods. Yet it is underdiagnosed and undertreated, even though it has a detrimental effect on almost all RA outcomes, including disease activity, arthritis-related complications, pain level, the chance of remission, quality of life, and mortality.

For these reasons, RA treatments aim to attain a better quality of life, which includes an active lifestyle, self-efficacy, mood stabilization, less pain (with non-catastrophizing, low intensity, without interference with everyday activities), increased activity, a good emotional and cognitive state, functional and psychological disability, adaptive coping styles (instead of learned helplessness), psychological balance, and work capability [22]. To achieve all these goals, appropriately integrating psychological interventions into interdisciplinary treatment is proven to be effective. Currently, it is scientifically supported that, despite pharmacological treatment, many RA patients still experience discomfort symptoms, such as psychological distress, pain, and fatigue [9], so it significantly affects their life. Furthermore, medicines for RA also have adverse drug effects, particularly when they are taken over long periods [1]. Therefore, complementary and alternative medicine therapies (including psychotherapies) are necessary for RA patients, even in the worst cases, without any long-term side effects [9]. As part of the complex treatment, psychological interventions can address the connection between thoughts and feelings that may help patients understand and emotionally accept the disease, which drives behavioral changes and better psychological function. In contrast, a single treatment by itself would not be effective for all these symptoms. These observations from the literature review and clinical practice helped develop our research questions. Precisely because of the fundamental complexity of rheumatoid arthritis, we considered it essential to examine which psychological methods and to what extent they are effective in treating RA, according to various well-controlled studies. Our work is, therefore, RA-specific, and we strived to include methodologically clear studies.

The statistical meta-analysis resulted in an overall maximum value of Hedges-g (Hedges-g = 0.856, Z = 4.223, *p* < 0.001), showing a significant effect size, and an overall average value of Hedges-g (Hedges-g = 0.399, Z = 0.399, *p* = 0.009), showing a medium effect size. These results further prove the psychological treatment of RA. Meanwhile, the overall minimum value of Hedges-g (Hedges-g = −0.047, Z = −0.335, *p* = 0.738) was insignificant, showing that psychological interventions in RA can be applied safely without adverse effects. Even in the worst case, the effect value was approximately 0, meaning that these methods are ineffective. On this basis, we can assume that, according to the studies in our meta-analysis, psychological interventions do not have any adverse effects. However, in the best case, these interventions can be highly effective. Moreover, these methods at least have moderate effects, even in average cases. All these results confirm previous findings that psychological interventions are essential in treating RA [21,23]. Based on the studies in this systematic review, we can confirm that psychological interventions positively affect RA patients, so their use is recommended.

Our research aligns with previous studies [1,2,22,44,47], which examined several psychological interventions in treating RA. Nevertheless, more studies are available on one specific psychological intervention [17,33,42], such as CBT or mindfulness [36,58]. Most studies show significant positive effects of psychological interventions [36,47,59]. As opposed to our study, in an overview of the literature, these studies often examine specific variables such as fatigue [22], self-efficacy [47], coping [35], or anxiety [24]. These significant results are, in many instances, contradictory [1] and require further distinction. In some cases, adverse effects are also available [60], which contrasts with our current results, which show that psychological interventions have no adverse effect, even in the worst case. To evaluate the efficacy of several types of psychological treatment, we conducted a series of comparative analyses in which the various psychological interventions did not show significant effect sizes. As we previously noted, it is difficult to isolate the specific psychological techniques because interventions such as CBT, patient education, and mindfulness utilize overlapping techniques derived from multiple theoretical backgrounds [2,23]. Therefore, our findings on the similarity of treatment effect sizes are unsurprising. Based on this, it is implied that no intervention type has been proven more effective than another.

The present study has several limitations and challenges that need to be acknowledged. First, it is important to note that the large effect size found for the overall interventions is essential. Although this cumulative effect size is significant, it does not directly mean that each psychological intervention works efficiently in all cases. A personalized combination of techniques is likely the most effective. Second, some studies included in this analysis barely described the techniques and theories applied in the intervention, which did not allow for the required accuracy during the coding process. Third, the studies included various outcome measures and populations, but most of the research involved middle-aged women. Furthermore, most of the overviewed articles need more low-quality evidence.

Several directions still need to be addressed in the present paper. Future research should identify which aspects of these psychological interventions cause long-term effects. In addition, future research should also examine the individual psychological interventions according to how many sessions and how long they lasted, exactly what kind of intervention took place, with what qualifications, and in what context it was conducted, whether it was an individual or group procedure, whether it took place in a team, or only multi modally. In RA treatments, finding only psychological interventions is quite rare. Thus, it is crucial to distinguish the articles depending on the combination of various interventions, such as psychological interventions with physical training, medical therapy, or social care. It is relevant to perform additional statistical analysis per variable and subgroup. Additionally, future longitudinal research should analyze the impact of the psychogenic factors of RA and the best combination of psychological interventions to maximize its considerable improvements and many benefits.

Unfortunately, a multidisciplinary approach to RA exists more in the literature than in practice. The relevant meta-analyses and systematic reviews state clearly that psychological treatments (relaxation, biofeedback, cognitive-behavioral therapy, mindfulness, hypnotherapy, relationship therapy, etc.) are just as effective for RA patients as for those with only mental disorders [21]. They should be used as adjuvant therapies for the disease. While the evidence for the effectiveness of methods that help cope with race, increase self-care capacity, modify emotional avoidance (dissociation), cure PTSD, expand spirituality, initiate post-traumatic growth, and involve family members is accumulating, it is yet to be explored how these protocols could be disseminated in clinical practice. It has been proven that various psychotherapy methods reduce the pain, anxiety, depression, and physical disability of RA patients [1,6,21]. Effective treatment of RA patients can only be achieved by a multidisciplinary professional team.

## 5. Conclusions

This study aimed to demonstrate the effectiveness of psychological interventions in treating RA. Since it has been proven that psychological interventions positively influence several dimensions of the condition of RA patients, their individualized and combined integration into the complex (biopsychosocial) therapy of RA is a medical task that cannot be postponed. Assuming that psychological interventions can influence physiological changes, our results further prove that psychological factors are closely associated with RA. Based on this logic, the two systems (physical and psychological) have shared mechanisms. Overall, there is substantial evidence that psychological interventions are moderately effective in the complex treatment of RA. Consequently, a multidisciplinary approach, including psychological interventions for RA treatment, is relevant and should be used as adjuvant therapy for the disease.

## Figures and Tables

**Table 1:** Effect sizes.

	Minimum Effect Sizes			Mean Effect Sizes			Maximum Effect Sizes		
	Estimate (Hedges-g)	Z	*p*	Estimate (Hedges-g)	Z	*p*	Estimate (Hedges-g)	Z	*p*
All interventions	−0.047	−0.335	0.738	0.399	2.61	0.009	0.856	4.223	<0.001
CBT	−0.318	−1.045	0.296	−0.349	−1.049	0.294	−0.378	−0.854	0.393
PE	0.174	0.617	0.537	0.180	0.583	0.560	0.144	0.350	0.726
R	0.239	0.559	0.576	0.466	0.952	0.341	0.835	1.357	0.175
M	0.116	0.270	0.787	−0.014	−0.029	0.977	−0.086	−0.137	0.891
ET	−0.263	−0.576	0.565	−0.433	−0.868	0.385	−0.615	−0.933	0.351
GT	−0.485	−0.771	0.441	−0.271	−0.392	0.695	−0.255	−0.278	0.781

Note. CBT: cognitive behavioral therapy; PE: patient education; R: relaxation techniques; M: mindfulness; ET: educational therapy; GT: group therapy.

## Data Availability

All data were obtained from published journal articles. The extracted data are available upon reasonable request to the corresponding author. This review was not registered, and no review protocol was prepared.

## Supplementary Materials

The following supporting information can be downloaded at: https://www.mdpi.com/article/10.3390/life13030849/s1, Table S1: List of rejected articles. Table S2: Summary Table: Study Characteristics.
