# Supplementary material for: The Effectiveness of Psychological Interventions for Rheumatoid Arthritis (RA): A Systematic Review and Meta-Analysis
Source: Life (Basel). 2023 Mar 21;13(3):849. doi: 10.3390/life13030849 (PMC10057722; doi:10.3390/life13030849)
Supplement: Supplementary file 1 [file life-13-00849-s001.zip › Supplementary Table S1.pdf]

Table S1. List of rejected articles

No.	File name	Final decision	In-text citation	Reason of rejection
**396**	A'yun_2019	No	A'yun & Hartini, 2019	Study design was not randomized controlled trial.
**1**	Abeson_2020	No	Abeson, 2020	Full text not available.
**2**	Abou_Raya_2014	No	Abou-Raya & Abou-Raya, 2014	Full text not available.
**397**	Abourazzak_2009	No	Abourazzak et al., 2009	Study design was not randomized controlled trial.
**3**	Ackerman_2016	No	Ackerman et al., 2016	Study design was not randomized controlled trial.
**398**	Akhlagi_2018	No	Akhlaghi et al., 2018	Necessary statistical data for meta-analysis was not provided.
**4**	Albano_2010	No	Albano et al., 2010	Study design was not randomized controlled trial.
**6**	Anderson_1985	No	Anderson et al., 1985	Study design was not randomized controlled trial.
**5**	Anderson_2004	No	Anderson et al., 2004	Study design was not randomized controlled trial.
**8**	APHA_1962	No	APHA, 1962	Study design was not randomized controlled trial.
**9**	APHA_1964	No	APHA, 1964	Study design was not randomized controlled trial.
**10**	APHA_1965	No	APHA, 1965	Study design was not randomized controlled trial.
**11**	APHA_1966	No	APHA, 1966	Study design was not randomized controlled trial.
**12**	APHA_1968	No	APHA, 1968	Study design was not randomized controlled trial.
**13**	APHA_1969	No	APHA, 1969	Study design was not randomized controlled trial.

**15**	Armour, G. (1950). Rheumatoid arthritis; physiotherapy and psychotherapy in spa treatment. Br J Phys Med, 13(1), 19-21.	No	Armour, 1950	Full text not available.
**17**	Astin_2002	No	Astin et al., 2002	Study design was not randomized controlled trial.
**18**	Bachmair_2021	No	Bachmair et al., 2021	Study population included non-RA patients.
**19**	Badgley_1971	No	Badgley et al., 1971	Study design was not randomized controlled trial.
**20**	Bagheri_Nesami_2006	No	Bagheri-Nesami et al., 2006	Study design was not randomized controlled trial.
**21**	Banerjee_2017	No	Banerjee et al., 2018	Study design was not randomized controlled trial.
**399**	Baratzadeh_2021	No	Baratzadeh et al., 2021	Written language was other than English.
**23**	Barlow_1997	No	Barlow et al., 1997	Intervention was not psychological therapy.
**24**	Barlow_1998a	No	Barlow et al., 1998	Study population included non-RA patients.
**25**	Barlow_1998b	No	Barlow et al., 1998	Study population included non-RA patients.
**26**	Barlow_2000	No	Barlow et al., 2000	Study population included non-RA patients.
**22**	Barlow_2009	No	Barlow et al., 2009	Study population included non-RA patients.
**29**	Basler_1991	No	Basler, 1991	Written language was other than English.
**28**	Basler_1993	No	Basler, 1993	Necessary statistical data for meta-analysis was not provided.
**30**	Bawa_2015	No	Bawa et al., 2015	Study design was not randomized controlled trial.
**32**	Bearne_2012	No	Bearne et al., 2012	Full text not available.
**31**	Bearne_2015	No	Bearne et al., 2015	Full text not available.
**33**	Beaulieu_2020a	No	Beaulieu et al., 2020	Full text not available.
**34**	Berg_1985	No	Berg et al., 1985	Necessary statistical data for meta-analysis was not provided.
**372**	Berman_2009	No	Berman et al., 2009	Study population included non-RA patients.

**36**	Bhattarai_2019	No	Bhattarai et al., 2019	Study population included non-RA patients.
**37**	Birch_2020	No	Birch et al., 2020	Study design was not randomized controlled trial.
**38**	Blaney_2021	No	Blaney et al., 2021	Study design was not randomized controlled trial.
**39**	Bode_2008	No	Bode et al., 2008	Study design was not randomized controlled trial.
**40**	Bos_2013	No	Bos et al., 2013	Study population included non-RA patients.
**41**	Brach_2010	No	Brach et al., 2010	Study design was not randomized controlled trial.
**44**	Bradley_1985	No	Bradley et al., 1985,	Full text not available.
**43**	Bradley_1999	No	Bradley & Alberts, 1999	Study design was not randomized controlled trial.
**42**	Bradley, L. A. (1989). Cognitive-behavioral therapy for primary fibromyalgia. J Rheumatol Suppl, 19, 131-136.	No	Bradley, 1989	Intervention was not psychological therapy.
**400**	Branch_1999	No	Branch et al., 1999	Study population included non-RA patients.
**47**	Broderick_2004	No	Broderick et al., 2004	Necessary statistical data for meta-analysis was not provided.
**48**	Bruce_1988	No	Bruce et al., 1988	Necessary statistical data for meta-analysis was not provided.
**49**	Brus_1997a	No	Brus et al., 1997	Study design was not randomized controlled trial.
**51**	Brycz_1988	No	Brycz, 1988	Written language was other than English.
**52**	Buckelew_1989	No	Buckelew & Parker, 1989	Study design was not randomized controlled trial.
**53**	Buckley_1990	No	Buckley et al., 1990	Study design was not randomized controlled trial.
**54**	Buszewicz_2006	No	Buszewicz et al., 2006	Study population included non-RA patients.
**55**	Cárdenas-Cloud_2021	No	Cárdenas-Cloud et al., 2021	Study design was not randomized controlled trial.

**56**	Carlson_2012	No	Carlson, 2012	Study design was not randomized controlled trial.
**57**	Carroll_1998	No	Carroll & Seers, 1998	Study design was not randomized controlled trial.
**58**	Carson_2006	No	Carson et al., 2006	Necessary statistical data for meta-analysis was not provided.
**59**	CDC_2009	No	Center for Disease Control & Prevention CDC, 2009	Study design was not randomized controlled trial.
**60**	CDC_2011	No	Center for Disease Control & Prevention CDC, 2011	Study design was not randomized controlled trial.
**61**	Cepeda_2006	No	Cepeda et al., 2006	Study design was not randomized controlled trial.
**62**	Chadi_2019	No	Chadi et al., 2019	Study population included non-RA patients.
**63**	Chambers_2000	No	Chambers & Schauenstein, 2000	Study design was not randomized controlled trial.
**64**	Chen_2021	No	Chen et al., 2021	Study population included non-RA patients.
**401**	Choi_2022	No	Choi et al., 2022	Study population included non-RA patients.
**65**	Chomistek_2020	No	Chomistek et al., 2020,	Study population included non-RA patients.
**66**	Chomsitek_2022	No	Chomistek et al., 2022	Study population included non-RA patients.
**67**	Chui_2004	No	Chui et al., 2004	Study design was not randomized controlled trial.
**68**	Cioppa_1975	No	Cioppa & Thal, 1975	Study design was not randomized controlled trial.
**69**	Clarke_1997	No	Clarke, 1997	Study design was not randomized controlled trial.
**70**	Clerget-Darpoux_2007	No	Clerget-Darpoux & Elston, 2007	Study design was not randomized controlled trial.
**402**	Connelly_2007	No	Connelly et al., 2007	Intervention was not psychological therapy.
**72**	Connelly_2019	No	Connelly et al., 2019	Study population included non-RA patients.
**73**	Conner_2006	No	Conner et al., 2006	Intervention was not psychological therapy.

**74**	Cook_2020	No	Cook et al., 2020	Full text not available.
**75**	Costa_2019	No	Costa et al., 2019	Study design was not randomized controlled trial.
**78**	Cramp_2013	No	Cramp et al., 2013	Study design was not randomized controlled trial.
**76**	Cramp_2019	No	Cramp, 2019	Study design was not randomized controlled trial.
**77**	Cramp, F., Hewlett, S., Almeida, C., Kirwan, J., Choy, E., Chalder, T., Pollock, J., & Christensen, R. (2011). Systematic review and meta-analyses of non-pharmacological interventions for fatigue in rheumatoid arthritis. Rheumatology, 50, iii99-iii100. https://www.cochranelibrary.com/central/doi/10.1002/central/CN-01003720/full	No	Cramp et al., 2011,	Full text not available.
**79**	Criswell, L. A., & Katz, P. P. (1994). Relationship of education level to treatment received for rheumatoid arthritis. J Rheumatol, 21(11), 2026-2033.	No	Criswell & Katz, 1994	Full text not available.
**80**	Ctri. (2019). Effect of mindfulness on Psychological well-being and functioning in Rheumatoid Arthritis.	No	Ctri, 2019	Unpublished clinical trial.

	https://trialsearch.who.int/Trial2.aspx?TrialID=CTRI/2019/12/022461. https://www.cochranelibrary.com/central/doi/10.1002/central/CN-02067144/full			
**81**	Cliss_2010	No	Cliss et al., 2010	Full text not available.
**82**	Dager_2012	No	Dager et al., 2012	Study design was not randomized controlled trial.
**373**	DanoffBurg_2006	No	Danoff-Burg et al., 2006	Study population included non-RA patients.
**84**	Davis_1990	No	Davis et al., 1990	Study design was not randomized controlled trial.
**87**	Davis_1994	No	Davis et al., 1994	Study design was not randomized controlled trial.
**85**	Davis_2006	No	Davis et al., 2006,	Intervention was not psychological therapy.
**445**	Davis_2008	No	Davis & White, 2008	Study design was not randomized controlled trial.
**88**	DeBrouwer_2011a	No	De Brouwer et al., 2011	Full text not available.
**89**	DeBrouwer_2013	No	De Brouwer et al., 2013	Other
**374**	deBuck_2005	No	De Buck et al., 2005	Intervention was not psychological therapy.
**91**	DeFreitas_2018	No	De Freitas, 2018	Study design was not randomized controlled trial.
**92**	DelRosso_2016	No	Del Rosso & Maddali-Bongi, 2016	Study design was not randomized controlled trial.
**403**	Deng_2013	No	Deng & Hu, 2013	Study design was not randomized controlled trial.
**375**	DeVellis_1988	No	Devellis et al., 1988	Necessary statistical data for meta-analysis was not provided.
**93**	DeVellis_1993	No	DeVellis & Blalock, 1993	Study design was not randomized controlled trial.

**94**	Dhokia_2020	No	Dhokia et al., 2020	Study population included non-RA patients.
**95**	DiRenzo_2018	No	DiRenzo & Finan, 2019	Study design was not randomized controlled trial.
**96**	DiRenzo_2019	No	DiRenzo et al., 2018	Study design was not randomized controlled trial.
**97**	Dissanayake_2010	No	Dissanayake & Bertouch, 2010	Study design was not randomized controlled trial.
**98**	Dixon_2007	No	Dixon et al., 2007	Study design was not randomized controlled trial.
**99**	Dixon-Woods_2007	No	Dixon-Woods et al., 2007	Study design was not randomized controlled trial.
**100**	Donovan_1991	No	Donovan, 1991	Study design was not randomized controlled trial.
**101**	Dowsey_2019	No	Dowsey et al., 2019	Study population included non-RA patients.
**102**	Dramsdahl_2019	No	Dramsdahl et al., 2019	Study population included non-RA patients.
**103**	Drks. (2019). Effectiveness and cost-effectiveness of guided internet-and mobile-based CBT for adolescents and young adults with chronic somatic conditions and comorbid depression and anxiety symptoms (youthCOACHcd): a multicentre randomized controlled trial with a 12-month follow-up. https://trialsearch.who.int/Trial2.aspx?TrialID=DRKS00017161. https://www.cochranelibra	No	Drks, 2019	Unpublished clinical trial.

	ry.com/central/doi/10.1002/ central/CN-02067289/full			
**104**	Drotar_2005	No	Drotar, 2005	Study design was not randomized controlled trial.
**105**	Duda_2010	No	Duda & Veldhuijzen van Zanten, 2010	Study design was not randomized controlled trial.
**106**	Dunne_2021	No	Dunne & Schubert, 2021	Study design was not randomized controlled trial.
**109**	Dures_2012	No	Dures et al., 2011,	Study design was not randomized controlled trial.
**107**	Dures_2014	No	Dures et al., 2012,	Study design was not randomized controlled trial.
**108**	Dures, E., Kitchen, K., Almeida, C., Ambler, N., Cliss, A., Hammond, A., & Knops, B. (2011). It's totally turned around the way I think: the patient perspective of cognitive behavioural therapy for fatigue in rheumatoid arthritis. Arthritis and rheumatism, 63(10). https://www.cochranelibrary.com/central/doi/10.1002/ central/CN-01005141/full	No	Dures et al., 2014	Full text not available.
**110**	Esbensen_2015	No	Esbensen, 2015	Study design was not randomized controlled trial.
**112**	Evers_1997	No	Evers et al., 1997,	Study design was not randomized controlled trial.
**113**	Evers_2001	No	Evers et al., 2001	Study design was not randomized controlled trial.
**404**	Evers_2003	No	Evers et al., 2003	Intervention was not psychological therapy.

**114**	Farah_2019	No	Farah et al., 2019	Study design was not randomized controlled trial.
**115**	Felsted_2020	No	Felsted, 2020	Study design was not randomized controlled trial.
**116**	Fenton_2020	No	Fenton et al., 2018	Full text not available.
**119**	Ferwerda_2016	No	Ferwerda et al., 2016,	Study population included non-RA patients.
**118**	Ferwerda_2018	No	Ferwerda et al., 2018	Other
**120**	Fiest_2017	No	Fiest et al., 2017	Study design was not randomized controlled trial.
**377**	Flor_1983	No	Flor et al., 1983	Study population included non-RA patients.
**121**	Fogarty_2015	No	Fogarty et al., 2015,	Full text not available.
**123**	Ford_2019	No	Ford & Long, 1977	Study design was not randomized controlled trial.
**124**	Freeman_2002	No	Freeman et al., 2002	Necessary statistical data for meta-analysis was not provided.
**405**	Garnefski_2013	No	Garnefski et al., 2013	Study population included non-RA patients.
**125**	Garza-Villareal_2014	No	Garza-Villarreal et al., 2014	Intervention was not psychological therapy.
**378**	Gay_2002	No	Gay et al., 2002	Study population included non-RA patients.
**126**	Geirhos_2022	No	Geirhos et al., 2022	Study population included non-RA patients.
**127**	Geissner, E., Jungnitsch, G., & Schmitz, J. (1994). [Psychological treatment approaches in pain. A comparative study of therapies in patients with chronic polyarthritis]. Z Klin Psychol Psychopathol Psychother, 42(4), 319-338.	No	Geissner et al., 1994	Written language was other than English.
**128**	Gerber_1987	No	Gerber et al., 1987	Full text not available.
**129**	Gerlich_2020	No	Gerlich et al., 2020	Written language was other than English.
**130**	Germond_1993	No	Germond et al., 1993	Necessary statistical data for meta-analysis was not provided.

**131**	Giacobbi_2015	No	Giacobbi et al., 2015	Study design was not randomized controlled trial.
**132**	Gillis_2006	No	Gillis et al., 2006	Study population included non-RA patients.
**406**	Goeppinger_1989	No	Goeppinger et al., 1989	Study population included non-RA patients.
**135**	Gronning_2012	No	Grønning et al., 2012,	Study population included non-RA patients.
**134**	Gronning_2014	No	Grønning et al., 2014	Study population included non-RA patients.
**136**	Guétin_2012	No	Guétin et al., 2012	Study population included non-RA patients.
**137**	Hammond_1998	No	Hammond, 1998,	Study design was not randomized controlled trial.
**408**	Hammond_1999	No	Hammond et al., 1999	Necessary statistical data for meta-analysis was not provided.
**140**	Hammond_2001	No	Hammond & Freeman, 2001,	Other
**138**	Hammond_2004	No	Hammond, 2004	Study design was not randomized controlled trial.
**409**	Hammond_2005	No	Hammond & Klompenhouwer, 2005	Study design was not randomized controlled trial.
**139**	Hammond_2008	No	Hammond et al., 2008	Study population included non-RA patients.
**142**	Harrold_2018	No	Harrold et al., 2018	Intervention was not psychological therapy.
**143**	Hawley_1995	No	Hawley, 1995	Study design was not randomized controlled trial.
**144**	Hawtin_2011	No	Hawtin & Sullivan, 2011	Study design was not randomized controlled trial.
**145**	Haydu_1963	No	Haydu, 1963	Study design was not randomized controlled trial.
**146**	Heiberg_2002	No	Heiberg & Kvien, 2002	Study design was not randomized controlled trial.
**379**	Helewa_1991	No	Helewa et al., 1991	Intervention was not psychological therapy.
**147**	Helliwell_1999	No	Helliwell et al., 1999	Necessary statistical data for meta-analysis was not provided.
**410**	Helmes_2015	No	Helmes et al., 2015	Study population included non-RA patients.

**148**	Herschbach_2010	No	Herschbach et al., 2010	Study population included non-RA patients.
**153**	Hewlett_2010	No	Hewlett et al., 2010,	Full text not available.
**151**	Hewlett_2015	No	Hewlett et al., 2015,	Study design was not randomized controlled trial.
**149**	Hewlett_2019	No	Hewlett et al., 2019	Other
**154**	Hewlett, S. E., Ambler, N., Knops, B., Cliss, A., Almeida, C., Pope, D., & Hammond, A. (2010). Sustained reduction in fatigue impact in rheumatoid arthritis: RCT of cognitive behavioural therapy. Arthritis and rheumatism, 62, 1325-. https://doi.org/10.1002/art.29091	No	Hewlett et al., 2010	Full text not available.
**155**	Hill_1997	No	Hill et al., 2001	Study design was not randomized controlled trial.
**156**	Hill_2001	No	Hill, 1997	Necessary statistical data for meta-analysis was not provided.
**158**	Horton-hausknecht_2000	No	Horton & Mitzdorf, 1994	Study design was not randomized controlled trial.
**157**	Horton, J. R., & Mitzdorf, U. (1994). Clinical hypnosis in the treatment of rheumatoid arthritis. Psychologische Beitrage, 36(1-2), 205-212.	No	Horton-hausknecht et al., 2000	Written language was other than English.
**159**	Hoving_2014	No	Hoving et al., 2014	Study design was not randomized controlled trial.

**164**	Irct20110514006480N. (2019). Effects of mindfulness-based stress reduction and cognitive behavioral therapy on patients with rheumatoid arthritis. https://trialsearch.who.int/Trial2.aspx?TrialID=IRCT20110514006480N18. https://www.cochranelibrary.com/central/doi/10.1002/central/CN-01948336/full	No	Unpublished clinical trial.
**161**	Irct2013051813363N. (2013). The effect of progressive musle relaxation on quality of life in rheumatoid arthritis patients. https://trialsearch.who.int/Trial2.aspx?TrialID=IRCT2013051813363N1. https://www.cochranelibrary.com/central/doi/10.1002/central/CN-01853331/full	No	Unpublished clinical trial.
**160**	Irct201308187531N. (2013). The effect of group education on self-management in patient with rheumatoid arthritis. https://trialsearch.who.int/Trial2.aspx?TrialID=IRCT201308187531N3.	No	Unpublished clinical trial.

	https://www.cochranelibrary.com/central/doi/10.1002/central/CN-01815266/full		
165	Irct20141012019511N. (2018). Efficacy of Cognitive-Behavioral Therapy Approach for Adherence and Depression (CBT-AD) on Depression and Non-Adherence to treatment. https://trialsearch.who.int/Trial2.aspx?TrialID=IRCT20141012019511N3. https://www.cochranelibrary.com/central/doi/10.1002/central/CN-01900490/full	No	Unpublished clinical trial.
162	Irct2015122425685N. (2016). The effect of self management program on elderly women with rheumatoid arthritis. https://trialsearch.who.int/Trial2.aspx?TrialID=IRCT2015122425685N1. https://www.cochranelibrary.com/central/doi/10.1002/central/CN-01870631/full	No	Unpublished clinical trial.
163	Irct2016022926846N. (2016). The effect of interventions based of psychological on the severity of insomnia and	No	Unpublished clinical trial.

	pain in rheumatoid arthritis. https://trialsearch.who.int/Trial2.aspx?TrialID=IRCT2016022926846N1. https://www.cochranelibrary.com/central/doi/10.1002/central/CN-01807984/full		
**166**	Irct20181128041778N. (2019). Effect of Mindfulness-based stress reduction and Cognitive behavior Therapy in Rheumatoid Arthritis Patients. https://trialsearch.who.int/Trial2.aspx?TrialID=IRCT20181128041778N1. https://www.cochranelibrary.com/central/doi/10.1002/central/CN-01972351/full	No	Unpublished clinical trial.
**167**	Isrctn. (2003). Education programmes for people with arthritis: a comparative study. https://trialsearch.who.int/Trial2.aspx?TrialID=ISRCTN11478835. https://www.cochranelibrary.com/central/doi/10.1002/central/CN-01798790/full	No	Unpublished clinical trial.
**168**	Isrctn. (2013). Reducing Arthritis Fatigue - clinical	No	Unpublished clinical trial.

	Teams using cognitive behavioural approaches (RAFT). https://trialsearch.who.int/Trial2.aspx?TrialID=ISRCTN52709998. https://www.cochranelibrary.com/central/doi/10.1002/central/CN-01805509/full			
**169**	Jamilian_2018	No	Jamilian et al., 2018	Full text not available.
**170**	Jeppesen_2012	No	Jeppesen et al., 2012	Full text not available.
**171**	Jeppesen_2013	No	Jeppesen et al., 2013	Full text not available.
**173**	Jolly_2018	No	Jolly et al., 2018	Study population included non-RA patients.
**174**	Kabat-Zinn_1982	No	Kabat-Zinn et al., 1985,	Study population included non-RA patients.
**175**	Kabat-Zinn_1985	No	Kabat-Zinn et al., 1992	Study population included non-RA patients.
**176**	Kabat-Zinn_1992	No	Kabat-Zinn, 1982	Study population included non-RA patients.
**446**	Kaplan_1981	No	Kaplan & Kozin, 1981	Full text not available.
**177**	Kaplan_1993	No	Kaplan & Kozin, 1981	Study population included non-RA patients.
**178**	Kaplan, S., & Kozin, F. (1981). A controlled study of group counseling in rheumatoid arthritis. J Rheumatol, 8(1), 91-99.	No	Kaplan et al., 1993	-
**179**	Katz_1998	No	Katz, 1998	Study design was not randomized controlled trial.
**380**	Keefe_1990a	No	Keefe et al., 1990	Study population included non-RA patients.
**381**	Keefe_1990b	No	Keefe et al., 1990	Study population included non-RA patients.
**182**	Keefe_1993	No	Keefe & Van Horn, 1993	Study design was not randomized controlled trial.
**382**	Keefe_1996	No	Keefe et al., 1996	Study population included non-RA patients.

**181**	Keefe_1997	No	Keefe & Caldwell, 1997	Study design was not randomized controlled trial.
**183**	Kelley_1997	No	Kelley et al., 1997	Necessary statistical data for meta-analysis was not provided.
**186**	Klompenhouwer_1999	No	Klompenhouwer, 1999	Study design was not randomized controlled trial.
**187**	Knittle_2010	No	Knittle et al., 2010	Study design was not randomized controlled trial.
**188**	Knittle_2011	No	Knittle et al., 2011	Intervention was not psychological therapy.
**189**	Knudson_1981	No	Knudson et al., 1981	Study design was not randomized controlled trial.
**190**	Koulil_2018	No	Koulil et al., 2018	Study design was not randomized controlled trial.
**191**	Kowalczewska, J. (1961). [Role of psychotherapy in the treatment of rheumatoid arthritis in young girls]. Reumatol Pol, 4, 65-76.	No	Kowalczewska, 1961	Written language was other than English.
**193**	Krawitz, M., & Wolman, T. (1979). Group therapy in rheumatoid arthritis. Pa Med, 82(12), 35-37.	No	Krawitz & Wolman, 1979	Full text not available.
**194**	Kuijper_2018	No	Kuijper et al., 2018	Intervention was not psychological therapy.
**195**	Kunzler_2020	No	Kunzler et al., 2020	Study design was not randomized controlled trial.
**196**	Kyngas_2003	No	Kyngäs, 2003	Study design was not randomized controlled trial.
**197**	Lacaille_2015	No	Lacaille et al., 2015	Full text not available.
**384**	Laforest_2008	No	Laforest et al., 2008	Study population included non-RA patients.
**411**	Laforest_2012	No	Laforest et al., 2012	Study population included non-RA patients.
**198**	Lahiri_2018	No	Lahiri et al., 2018	Full text not available.
**412**	Lahiri_2020	No	Lahiri et al., 2022	Intervention was not psychological therapy.

**199**	Lalloo_2021	No	Lalloo et al., 2021	Study population included non-RA patients.
**200**	Lange_1995	No	Lange, 1995	Study design was not randomized controlled trial.
**201**	Langer, H. E. (1995). [Patient education--a contribution to improvement of long-term management of patients with rheumatism]. Z Rheumatol, 54(4), 207-212.	No	Langer, 1995	Written language was other than English.
**202**	Langer, H. E., & Birth, U. (1987). [Patient education in chronic polyarthritis. 2. Organizational and curriculum concept of patient seminars for chronic polyarthritis patients]. Z Rheumatol, 46(6), 333-338.	No	Langer & Birth, 1987,	Written language was other than English.
**203**	Langer, H. E., & Birth, U. (1988). [Patient education in chronic polyarthritis. 3. Intermediate results of a prospective, controlled study of the effectiveness and side effects of patient seminars for polyarthritis patients]. Z Rheumatol, 47(1), 43-51.	No	Langer & Birth, 1988	Written language was other than English.
**204**	Langer, H. E., & Mattussek, S. (1990). [Patient education in rheumatology]. Wien Med	No	Langer & Mattussek, 1990	Written language was other than English.

	Wochenschr, 140(12), 349-351.			
**205**	Latocha_2020	No	Latocha et al., 2020	Necessary statistical data for meta-analysis was not provided.
**206**	Lavigne_1992	No	Lavigne et al., 1992	Study population included non-RA patients.
**207**	le Gallez, P. (1984). Patient education and self-management. Nursing (Lond), 2(31), 916-917.	No	le Gallez, 1984	Full text not available.
**208**	LeFort_2001	No	LeFort, 2001	Full text not available.
**209**	Lehew_1970	No	Lehew, 1970	Study design was not randomized controlled trial.
**210**	Leibing_1999	No	Leibing et al., 1999	Necessary statistical data for meta-analysis was not provided.
**413**	Lempp_2017	No	Lempp et al., 2017	Necessary statistical data for meta-analysis was not provided.
**211**	Leverone_2010	No	Leverone & Epstein, 2010	Study design was not randomized controlled trial.
**414**	Li_2005	No	Li et al., 2005	Intervention was not psychological therapy.
**212**	Li_2017	No	Li et al., 2017	Study population included non-RA patients.
**415**	Li_2021	No	Li et al., 2021	Intervention was not psychological therapy.
**385**	Lin_2003	No	Lin et al., 2003	Study population included non-RA patients.
**213**	Lindberg_1988	No	Lindberg & Lindberg, 1988	Study design was not randomized controlled trial.
**214**	Lindberg, N. E., & Lindberg, E. (1998). [Psychotherapy can be effective in rheumatoid arthritis]. Lakartidningen, 95(28-29), 3162-3163.	No	Lindberg & Lindberg, 1998	Written language was other than English.
**215**	Lindberg, N. E., Lindberg, E., Theorell, T., & Larsson, G. (1996). Psychotherapy	No	Lindberg et al., 1996	Written language was other than English.

	in rheumatoid arthritis--a parallel-process study of psychic state and course of rheumatic disease. Z Rheumatol, 55(1), 28-39.			
**386**	Lindroth_1997	No	Lindroth et al., 1997	Necessary statistical data for meta-analysis was not provided.
**216**	Logan_2012	No	Logan & Magem, 2012	Full text not available.
**416**	Logan_2012	No	Logan & Magem, 2012	Full text not available.
**217**	Lomholt_2015	No	Lomholt et al., 2015	Study population included non-RA patients.
**218**	Lopez-Olivio_2015	No	Lopez-Olivo et al., 2015	Full text not available.
**219**	Lopez-Olivio_2021	No	Lopez-Olivo et al., 2021	Intervention was not psychological therapy.
**222**	Lorig_1982	No	Lorig, 1982	Study design was not randomized controlled trial.
**387**	Lorig_1989	No	Lorig et al., 1989	Study population included non-RA patients.
**220**	Lorig_1993	No	Lorig & Holman, 1993	Study design was not randomized controlled trial.
**447**	Lorig_1999	No	Lorig et al., 1999	Study population included non-RA patients.
**223**	Lorig_2004	No	Lorig et al., 2004	Study population included non-RA patients.
**221**	Lorig_2005	No	Lorig et al., 2005	Study population included non-RA patients.
**224**	Lorig_2008	No	Lorig et al., 2008	Study population included non-RA patients.
**226**	Lumley_2012	No	Lumley et al., 2012	Study design was not randomized controlled trial.
**418**	Lumley_2018	No	Lumley et al., 2018	Other
**228**	Lundgren_1996	No	Lundgren & Stenström, 1996	Full text not available.
**227**	Lundgren_1999	No	Lundgren & Stenström, 1999	Necessary statistical data for meta-analysis was not provided.
**229**	Lunkenheimer_2020	No	Lunkenheimer et al., 2020	Study population included non-RA patients.

**230**	MacIver_2021	No	MacIver et al., 2021	Study design was not randomized controlled trial.
**231**	MacPherson_2013	No	MacPherson et al., 2013	Study population included non-RA patients.
**232**	Maisiak_1996	No	Maisiak et al., 1996	Study population included non-RA patients.
**233**	Makelainen_2009	No	Mäkeläinen et al., 2009	Study design was not randomized controlled trial.
**234**	Malysheva_2015	No	Malysheva et al., 2015	Intervention was not psychological therapy.
**235**	Management_2004	No	Management, 2004	Full text not available.
**236**	Manning_2013	No	Manning et al., 2013	Full text not available.
**237**	Marchal, J. (1970). [Efficacy of mental imagery, used as therapeutic method in psychosomatic medicine]. Riv Sper Freniatr Med Leg Alien Ment, 94(5), 1292-1309.	No	Marchal, 1970	Written language was other than English.
**238**	Markozannes_2017	No	Markozannes et al., 2017	Study design was not randomized controlled trial.
**239**	Marques_2021	No	Marques et al., 2021	Study population included non-RA patients.
**241**	Martin_2017	No	Martin et al., 2017	Intervention was not psychological therapy.
**240**	Martin_2019	No	Martin et al., 2019	Study population included non-RA patients.
**448**	Mathieux_2009	No	Mathieux et al., 2009	Intervention was not psychological therapy.
**242**	Mayoux-Benhamou_2008	No	Mayoux-Benhamou et al., 2008	Necessary statistical data for meta-analysis was not provided.
**243**	McCarron_2015	No	McCarron, 2015	Study design was not randomized controlled trial.
**244**	McCracken, L. M. (1991). Cognitive-behavioral treatment of rheumatoid arthritis: A preliminary review of efficacy and	No	McCracken, 1991	Full text not available.

	methodology. Annals of Behavioral Medicine, 13(2), 57-65.			
**245**	Mehta_2019	No	Mehta et al., 2019	Study design was not randomized controlled trial.
**246**	Mendelson_2011	No	Mendelson et al., 2011	Study population included non-RA patients.
**247**	Menefee_2014	No	Menefee, 1994	Study design was not randomized controlled trial.
**420**	Menzies_2022	No	Menzies et al., 2022	Necessary statistical data for meta-analysis was not provided.
**248**	Merkes_2010	No	Merkes, 2010	Study design was not randomized controlled trial.
**421**	Michou_2022	No	Michou et al., 2022	Necessary statistical data for meta-analysis was not provided.
**249**	Miller_1995	No	Miller et al., 1995	Study population included non-RA patients.
**250**	Mollard_2018	No	Mollard & Michaud, 2018	Necessary statistical data for meta-analysis was not provided.
**423**	Momeni_2020	No	Momeni et al., 2020	Written language was other than English.
**424**	Mooney_2012	No	Mooney, 2012	Necessary statistical data for meta-analysis was not provided.
**251**	Mullen, P. D., Laville, E. A., Biddle, A. K., & Lorig, K. (1987). Efficacy of psychoeducational interventions on pain, depression, and disability in people with arthritis: a meta-analysis. J Rheumatol Suppl, 14 Suppl 15, 33-39.	No	Mullen et al., 1987	Full text not available.
**252**	Mulligan_2003	No	Mulligan & Newman, 2003	Study design was not randomized controlled trial.
**254**	Murphy_2014	No	Murphy et al., 2014	Study population included non-RA patients.

**425**	Nazemi_2018	No	Nazemi et al., 2018	Study design was not randomized controlled trial.
**255**	Nct. (2003). Coping Skills Training for Early Rheumatoid Arthritis. https://clinicaltrials.gov/show/NCT00056394. https://www.cochranelibrary.com/central/doi/10.1002/central/CN-01509211/full	No	Nct, 2003	Unpublished clinical trial.
**256**	Nct. (2010). Psychosocial Treatment Intervention in Persons Newly Diagnosed With Rheumatoid Arthritis or Diabetes. https://clinicaltrials.gov/show/NCT01066130. https://www.cochranelibrary.com/central/doi/10.1002/central/CN-01528064/full	No	Nct, 2010	Unpublished clinical trial.
**257**	Ndosi_2016	No	Ndosi et al., 2016	Intervention was not psychological therapy.
**258**	Neill_2006	No	Neill et al., 2006	Study design was not randomized controlled trial.
**259**	Newman_2004	No	Newman et al., 2004	Study design was not randomized controlled trial.
**260**	Nguyen_2021	No	Nguyen et al., 2021	Study population included non-RA patients.
**427**	Nordmark_2006	No	Nordmark et al., 2006	Intervention was not psychological therapy.
**261**	Ntr. (2007). Psychophysiological stress mechanisms in chronic inflammatory diseases. https://trialsearch.who.int/Trial2.aspx?TrialID=NTR11	No	Ntr, 2007	Unpublished clinical trial.

	93. https://www.cochranelibrary.com/central/doi/10.1002/central/CN-01825599/full			
262	Ntr. (2009). Tailored cognitive-behavioral E-health care in patients with rheumatoid arthritis. https://trialsearch.who.int/Trial2.aspx?TrialID=NTR2100. https://www.cochranelibrary.com/central/doi/10.1002/central/CN-01863832/full	No	Ntr, 2009	Unpublished clinical trial.
263	Ntr. (2012). Effect of mindfulness on patients with rheumatoid arthritis: A controlled effect study. https://trialsearch.who.int/Trial2.aspx?TrialID=NTR3458. https://www.cochranelibrary.com/central/doi/10.1002/central/CN-01868647/full	No	Ntr, 2012	Unpublished clinical trial.
264	Ntr. (2012). Goal management training for patients with arthritis. https://trialsearch.who.int/Trial2.aspx?TrialID=NTR3606. https://www.cochranelibrary.com/central/doi/10.1002/central/CN-01868982/full	No	Ntr, 2012	Unpublished clinical trial.

**265**	O'Leary_1988	No	O’Leary et al., 1988	Necessary statistical data for meta-analysis was not provided.
**429**	Osborne_2007	No	Osborne et al., 2007	Study population included non-RA patients.
**266**	Otop, J. (1978). [Psychotherapy in the rehabilitation of patients with rheumatoid arthritis]. Wiad Lek, 31(2), 135-137.	No	Otop, 1978	Written language was other than English.
**267**	Ottonello_2007	No	Ottonello, 2007	Study design was not randomized controlled trial.
**268**	Panagopoulou_2006	No	Panagopoulou et al., 2006	Study design was not randomized controlled trial.
**269**	Parker_1988	No	Parker et al., 1988	Necessary statistical data for meta-analysis was not provided.
**270**	Parker_1993	No	Parker et al., 1993	Study design was not randomized controlled trial.
**272**	Parker_1995	No	Parker et al., 1995	Necessary statistical data for meta-analysis was not provided.
**273**	Parker_2003	No	Parker et al., 2003	Necessary statistical data for meta-analysis was not provided.
**271**	Parker, J. C., Singsen, B. H., Hewett, J. E., Walker, S. E., Hazelwood, S. E., Hall, P. J., Holsten, D. J., & Rodon, C. M. (1984). Educating patients with rheumatoid arthritis: a prospective analysis. Arch Phys Med Rehabil, 65(12), 771-774.	No	Parker et al., 1984	Full text not available.
**274**	Parlar_2013	No	Parlar et al., 2013	Study design was not randomized controlled trial.
**275**	Pei_2020	No	Pei et al., 2021	Study design was not randomized controlled trial.

**276**	Plummer_2016	No	Plummer et al., 2016	Full text not available.
**277**	Podgorski_1985	No	Podgorski & Edmonds, 1985	Study design was not randomized controlled trial.
**430**	Pot-Vaucel_2016	No	Potts & Brandt, 1983	Necessary statistical data for meta-analysis was not provided.
**278**	Potts_1983	No	Pot-Vaucel et al., 2016	Necessary statistical data for meta-analysis was not provided.
**279**	Poulsen_1991	No	Poulsen, 1991	Study design was not randomized controlled trial.
**280**	Poulsen, A., Tvede, N., & Egidius, L. (1988). [The effect of multidisciplinary group advice for patients with rheumatoid arthritis]. Ugeskr Laeger, 150(49), 3047-3050.	No	Poulsen et al., 1988	Written language was other than English.
**282**	Prothero_2018	No	Prothero et al., 2018	Study design was not randomized controlled trial.
**431**	Prothero_2020	No	Prothero, 2020	Study design was not randomized controlled trial.
**283**	Racaru_2021	No	Racaru et al., 2021	Study design was not randomized controlled trial.
**432**	Radhamaniamma_2013	No	Radhamaniamma et al., 2013	Intervention was not psychological therapy.
**284**	Radner_2012	No	Radner et al., 2012	Study design was not randomized controlled trial.
**286**	RaunsbaekKnudsen_2021	No	Raunsbaek Knudsen et al., 2021	Necessary statistical data for meta-analysis was not provided.
**433**	Reid_2013	No	Reid et al., 2014	Study population included non-RA patients.
**287**	Reiff, H. (1988). [Rheumatoid arthritis: reconstruction of the body image exemplified by	No	Reiff, 1988	Written language was other than English.

	psychoanalytic therapy]. Psychother Psychosom Med Psychol, 38(8), 282-287.			
**288**	Rhee_2000	No	Rhee et al., 2000	Necessary statistical data for meta-analysis was not provided.
**289**	Richards_2012a	No	Richards et al., 2012	Study design was not randomized controlled trial.
**290**	Richards_2012b	No	Richards et al.,2012	Study design was not randomized controlled trial.
**292**	Riemsma_2002	No	Riemsma et al., 2002	Study design was not randomized controlled trial.
**291**	Riemsma_2003a	No	Riemsma et al., 2003	Study design was not randomized controlled trial.
**294**	Rodríguez-Sánchez-Laulhé_2020	No	Rodríguez-Sánchez-Laulhé et al., 2020	Necessary statistical data for meta-analysis was not provided.
**295**	Rogers_1982	No	Rogers et al., 1982	Study design was not randomized controlled trial.
**434**	Ronen_1996	No	Ronen et al., 1996	Study population included non-RA patients.
**296**	Rzeszutek_2017	No	Rzeszutek et al., 2017	Study design was not randomized controlled trial.
**297**	Salmon_2015	No	Salmon et al., 2015	Full text not available.
**298**	Salmon_2019	No	Salmon et al., 2019	Study design was not randomized controlled trial.
**299**	Santos_2018	No	Santos et al., 2018	Study design was not randomized controlled trial.
**300**	Santos_2019	No	Santos et al., 2019	Study design was not randomized controlled trial.
**301**	Saperia_2012	No	Saperia & Swartzman, 2012	Study design was not randomized controlled trial.
**302**	Savelkoul_2003	No	Savelkoul et al., 2003	Study design was not randomized controlled trial.

**304**	Schwartz_1978	No	Schwartz et al., 1978	Study design was not randomized controlled trial.
**303**	Schwartz_2017	No	Schwartz et al., 2017	Study design was not randomized controlled trial.
**306**	Shafii_1973	No	Shafii, 1973	Study design was not randomized controlled trial.
**307**	Shao_2020	No	Shao et al., 2020	Study design was not randomized controlled trial.
**308**	Shao_2021	No	Shao et al., 2021	Necessary statistical data for meta-analysis was not provided.
**309**	Shariff_2009	No	Shariff et al., 2009	Study design was not randomized controlled trial.
**312**	Sharpe_2008	No	Sharpe et al., 2008	Necessary statistical data for meta-analysis was not provided.
**310**	Sharpe_2013	No	Sharpe, 2013	Study design was not randomized controlled trial.
**311**	Sharpe_2016	No	Sharpe, 2016	Study design was not randomized controlled trial.
**317**	Shen_2020	No	Shen et al., 2020	Study design was not randomized controlled trial.
**318**	Shigaki_2008	No	Shigaki et al., 2008	Necessary statistical data for meta-analysis was not provided.
**320**	Siedliecki_2006	No	Siedliecki & Good, 2006	Intervention was not psychological therapy.
**323**	Sinclair_1998	No	Sinclair & Scroggie, 2005	Necessary statistical data for meta-analysis was not provided.
**322**	Sinclair_2001	No	Sinclair & Wallston, 2001	Necessary statistical data for meta-analysis was not provided.
**321**	Sinclair_2005	No	Sinclair et al., 1998	Study population included non-RA patients.
**435**	Smarr_1997	No	Smarr et al., 1997	Necessary statistical data for meta-analysis was not provided.
**325**	Smarr_2011	No	Smarr et al., 2011	Necessary statistical data for meta-analysis was not provided.

**324**	Smarr, K., Musser, D., Hanson, K., Laffey, J., Johnson, R., Siva, C., & Parker, J. (2005). Development of an online self-management intervention for persons with rheumatoid arthritis: The RAhelp. org Project. Arthritis and rheumatism,	No	Smarr et al., 2005	Full text not available.
**390**	Smyth_1998	No	Smyth, 1998	Study design was not randomized controlled trial.
**326**	Solomon_2002	No	Solomon et al., 2002	Study population included non-RA patients.
**327**	Somers_2010	No	Somers et al., 2010	Study design was not randomized controlled trial.
**328**	Southwood_1995	No	Southwood, 1995	Study design was not randomized controlled trial.
**329**	Spijk-deJonge_2021	No	Spijk-de Jonge et al., 2021	Study design was not randomized controlled trial.
**330**	Spilberg, F. (1984). A pain management treatment for rheumatoid arthritis patients: Stress inoculation training for pain and cognitive therapy for depression. 1925-1925.	No	Spilberg, 1984	Full text not available.
**331**	Srikesavan_1984	No	Srikesavan et al., 2019	Study design was not randomized controlled trial.
**333**	Stinson_2010	No	Stinson et al., 2010	Study population included non-RA patients.
**332**	Stinson_2020	No	Stinson et al., 2020	Study population included non-RA patients.
**334**	Stinson, J. N., McGrath, P. J., Hodnett, E. E., Feldman, B., Duffy, C. M., Huber, A.,	No	Stinson et al., 2009	Full text not available.

	& Tucker, L. B. (2009). Feasibility testing of an online self-management program for adolescents with juvenile idiopathic arthritis (JIA): a pilot randomized controlled trial. Arthritis and rheumatism, 60, 238-. https://doi.org/10.1002/art.25321			
**392**	Stone_2000	No	Stone et al., 2000	Study population included non-RA patients.
**335**	Strating_2006	No	Strating et al., 2006	Intervention was not psychological therapy.
**336**	Strauss_1986	No	Strauss et al., 1986	Necessary statistical data for meta-analysis was not provided.
**338**	Taal_1993	No	Taal et al., 1993	Necessary statistical data for meta-analysis was not provided.
**337**	Taal_1997	No	Taal et al., 1997	Study design was not randomized controlled trial.
**339**	Tam_2019	No	Tam et al., 2019	Necessary statistical data for meta-analysis was not provided.
**340**	Test_2020	No	Test, 2020	Study design was not randomized controlled trial.
**341**	Thabane_2010	No	Thabane et al., 2010	Study design was not randomized controlled trial.
**342**	Thomsen_2020	No	Thomsen et al., 2020	Necessary statistical data for meta-analysis was not provided.
**343**	Tiliakos_2011	No	Tiliakos et al., 2011	Other
**344**	Torem_2007	No	Torem, 2007	Study design was not randomized controlled trial.
**438**	Trudeau_2015	No	Trudeau et al., 2015	Study population included non-RA patients.
**345**	Udelman_1978	No	Udelman & Udelman, 1978	Study design was not randomized controlled trial.

**439**	vanCranenburgh_2015	No	van Cranenburgh, 2016	Study design was not randomized controlled trial.
**393**	vanLankveld_2004	No	van Lankveld et al., 2004	Study design was not randomized controlled trial.
**348**	vanMiddendorp_2007	No	van Middendorp et al., 2007	Study design was not randomized controlled trial.
**346**	vanMiddendorp_2008	No	van Middendorp & Geenen, 2008	Necessary statistical data for meta-analysis was not provided.
**349**	VanUden-Kraan_2011	No	Van Uden-Kraan et al., 2011	Study design was not randomized controlled trial.
**350**	Veitiene_2005	No	Veitiene & Tamulaitiene, 2005	Study design was not randomized controlled trial.
**352**	ViletVlieland_1996	No	Vliet Vlieland et al., 1996	Necessary statistical data for meta-analysis was not provided.
**351**	ViletVlieland_2011	No	Vliet Vlieland & van den Ende, 2011	Study design was not randomized controlled trial.
**440**	Vriezekolk_2013	No	Vriezekolk et al., 2013	Study population included non-RA patients.
**353**	Walco_1992	No	Walco et al., 1992	Study population included non-RA patients.
**354**	Walker_2007	No	Walker et al., 2007	Intervention was not psychological therapy.
**355**	Walrabenstein_2021	No	Walrabenstein et al., 2021	Necessary statistical data for meta-analysis was not provided.
**441**	Walrabenstein_2021	No	Walrabenstein et al., 2021	Necessary statistical data for meta-analysis was not provided.
**442**	Wells-Federman_2002	No	Wells-Federman et al., 2002	Study population included non-RA patients.
**357**	Williams_2020	No	Williams et al., 2020	Study design was not randomized controlled trial.
**358**	Wiskin_1998	No	Wiskin, 1998	Study design was not randomized controlled trial.
**359**	Wu_2022	No	Wu et al., 2022	Study design was not randomized controlled trial.
**360**	Ying_2016	No	Ying et al., 2016	Full text not available.

**394**	Young_1995	No	Young et al., 1995	Necessary statistical data for meta-analysis was not provided.
**449**	Zangi_2011	No	Zangi et al., 2012	Study population included non-RA patients.
**361**	Zautra_2005	No	Zautra et al., 2005	Study design was not randomized controlled trial.
**363**	Zautra_2007	No	Zautra et al., 2007	Intervention was not psychological therapy.
**365**	Zhou_2020	No	Zhou et al., 2020	Study design was not randomized controlled trial.
**367**	Zuidema_2015	No	Zuidema et al., 2015	Study design was not randomized controlled trial.
**368**	Zuidema_2019b	No	Zuidema et al., 2019	Study design was not randomized controlled trial.
**369**	Zwikker_2012	No	Zwikker et al., 2012	Study design was not randomized controlled trial.
