# Supplementary material for: The Effectiveness of Psychological Interventions for Rheumatoid Arthritis (RA): A Systematic Review and Meta-Analysis
Source: Life (Basel). 2023 Mar 21;13(3):849. doi: 10.3390/life13030849 (PMC10057722; doi:10.3390/life13030849)
Supplement: Supplementary file 1 [file life-13-00849-s001.zip › Supplementary Table S2.pdf]

Table S2. Summary table: Study Characteristics

*UD= undisclosed

Name of file	References	Source of sample	Exclusion criteria	Total participants (drop out)		Female%		Mean age (SD)		Average duration of diagnosis	Intervention (s)	Control condition	Duration of intervention	Assessment	Outcome measures	Results
				Experimental	Control	Experimental	Control	Experimental	Control							
Anvar_2018	Anvar et al, 2018 [2]	Women who were referred to the rheumatology clinic in the city of Tabriz in northwest of Iran.	cognitive impairment, frail to undertake the research	40(3)	40(1)	100	100	UD*	UD	UD	Arthritis Self-Management Program (ASMP)	routine medical management	6 weeks	1) baseline 2) 4 - 5 months	Arthritis Self-Efficacy Scale (ASES)	Significant increases were found for self-efficacy pain scale in the intervention group compared to the control group (p=0.000).

Appelbaum_1988	Appelbaum et al, 1988 [3]	All patients were from Albany, New York, Veterans Administration Medical Center	not meeting the criteria defined by the Arthritis Foundation (1981)	9(0)	9(0)	11,11	11,11	61.9(9.2)	62.6(8.8)	9-20 year	Cognitive-Behavioural Pain Management, Relaxation training, Biofeedback	routine medical management	6 weeks	1) baseline 2) post-intervention 3) 18 months	Weekly Arthritis Diary (WAD), McGill Pain Questionnaire (MPQ), Daily Activities Questionnaire (DAQ)	There were significant intercorrelations on WAD measures concerned with pain report and with sleep parameters. Significant pre-post effects for weekly pain index, weekly peak pain rating, and morning stiffness were noted.

Arvidsson_2012	Arvidsson et al, 2013 [4]	All patients were from a rheumatology unit in the southwest of Sweden.	lack of knowledge of the Swedish language	38(0)	124(0)	0,71	0,73	56. 4(7.2)	55.2 (13.2)	RA<1 year	Self-management, Education	routine medical management	1 year	1) baseline 2) 1 wk 3) 6 months	Short Form-36 Health Survey (SF-36), Swedish Rheumatic Disease Empowerment Scale (SWE-RES-23), Appraisal of Self-Care Agency Scale (ASA-A),Sense of Coherence (SOC)	The experimental group scored statistically significant improvement between baseline and 6-month post-intervention in waking during the night (P = 0046) and feeling fatigue the last week (P = 0048). In the control group, there was a statistically significant deterioration between 1-week post-intervention and 6-month post-intervention in feeling rested after sleep (P = 0. 0048).
Barsky_2010	Barsky et al, 2010 [5]	Most subjects were identified through the hospital's	fibromyalgia, serious medical comorbidity	68(0)<br>44(0)	56(0)<br>56(0)	90<br>82	87<br>87	54.3 (13.1)<br>54.0 (12.3)	51.9 (13.4)<br>51.9 (13.4)	13 years since RA	CBT<br>Relaxation training	Arthritis Education	8- to 12-week	1) baseline 2) 12 months	Rheumatoid Arthritis Symptom Questionnair	Significant benefits were found for pain, other RA

computerized patient registry, which was queried for all patients with a diagnosis of RA. A smaller number of subjects volunteered for the study in response to public announcements and advertisements.	diagnosis	e (RASQ), Arthritis Impact Measurement Scale (AIMS)	symptoms, self-care activities, and social activities.

Bernateck_2008	Bernateck et al, 2008 [6]	Participants were recruited by the outpatient clinic of the Department of Rheumatology, Hannover Medical School, or through announcements in local newspapers.	receiving systemic corticoid treatment >7.5 mg/day prednisolone, tumor necrosis factor alpha (TNF-α) inhibitors, transcutaneous electrical nerve stimulation (TENS) or physiotherapy, a new pharmacological treatment for RA or analgesia in the last 8 weeks, receiving acupuncture treatment during the last 6 months, pregnancy or lactation, malignant or psychiatric diseases, therapy using a cardiac pulse generator	18(0)	19(0)	88,88	73,68	52.22 (11.19)	51.05 (13.18)	RA ≥6 months	Autogenic training	Adjuvant Auricular Electroacupuncture	6 weeks	1) baseline 2) post-intervention	Erythrocyte sedimentation rate (ESR),Tumour Necrosis Factor (TNF-α), Calcitonin gene related peptide (CGRP),Substance Interleukin 6 and 10	The adjuvant use of both EA and AT in the treatment of RA resulted in significant short- and long-term treatment effects in all outcome parameters.
Bradley_1987	Bradley, L. A., 1989 [7]	Participants were recruited from the Section on Rheumatology of	not meeting the criteria of American Rheumatism	17(0)	18(0)	UD	UD	47.65 (13.92)	50.50 (11.14)	11 years	Biofeedback-assisted, cognitive-behavioural	no adjunct treatment (NAT)	UD	1) post-intervention 2) 6	Visual Analogue Scale (VAS), Rheumatoid	Significant reductions were found in trait anxiety,

the Bowman Gray School of Medicine.	Association for a diagnosis of RA	18(0)	18(0)	UD	UD	52.00(12.51)	50.50 (11.14)	group therapy (CBT) Structured group social support therapy (SGT)		month follow up	Activity Index (RAI), Subject assessment of disease activity, Rheumatologist or nurse assessment of disease activity, Articular index, Grip strength, Rheumatoid factor tier , Erythrocyte sedimentation rate (ESR), State-Trait Anxiety Inventory (STAI),Depression Adjective Check List (DACL) , Change in skin temperature with /without biofeedback, Health Locus of Control (HLC), Arthritis Helplessness Index (AHI)	patients' pain behaviour and disease activity.

Breedland_2011	Breedland et al, 2011 [8]	Participants were recruited from a rehabilitation centre in the Netherlands	severe disease activity (Disease Activity Score [DAS-28], cardiac or pulmonary diseases resulting in restrictions in their ability to follow a physical exercise program, a Steinbrocker classification of functional capacity, no stable medication for the RA, intraarticular injections during the time of the study	13(0)	13(0)	UD	UD	45 (11.9)	51.8 (9.4)	10 years	The FIT program: physical exercise, educational component, improving self-efficacy	routine medical management	8 weeks	1) baseline 2) 9 wk	Vo2 max, Microfet, Arthritis Impact Measurement Scales - 2 (Dutch-AIMS2), Arthritis Self-Efficacy Scale (ASES)	The intervention group showed significant improvement in V̇O2max and also significant within-group changes were found over time for muscle strength of the upper and lower extremities and health status.
Brus_1997b	Brus et al, 1997 [9]	UD	had not entered the practices of 3 rheumatologists less than 8 years ago	7(0)<br>25(0)	6(0)<br>22(0)	UD<br>UD	UD<br>UD	51.6 (9.3)<br>50.2 (10.8)	50.2 (11.2)<br>49.0 (11.0)	5-8 years<br>3-4 years	Patient education	ESR > 28<br>ESR < 28	4 months	1) baseline 2) 4 months	Physical, endurance and relaxation exercises, Arthritis Impact Measuremen	There were no significant differences between the adherence parameters of the various

and not fulfilled the American College of Rheumatology criteria for RA				t Scales (Dutch-AIMS), Modified Health Assessment Questionnaire (M-HAQ	pairs of groups.

Conn_2013	Conn et al, 2013 [10]	Participants were RA patients meeting entrance criteria from the Grady Hospital Arthritis Clinic in Atlanta, GA, USA. Most were African American and financially disadvantaged.	limited mental capacity from a congenital brain disorder, psychosis, depression, drug dependency, uncontrolled chronic diseases, chronic lung disease, uncontrolled congestive heart failure, stroke, stage renal disease, sickle cell anemia, HIV–AIDS, insulin-dependent complicated diabetes mellitus	40(12)	34(18)	28703	28703	54.2 (8.2)	52.9 (10.2)	6-7 years	Arthritis self-management program	routine medical management	18 months	1) baseline 2) 6 months 3)12 month 4)18 month	Swollen joint count, Tender joint count, Health Assessment Questionnaire (HAQ)	There were no significant differences between the groups, excepted the swollen joint count (p=0.02)

Dalili_2019	Dalili et al, 2011 [11]	RA patients, who visited the clinic of Jam Rheumatology Centres and met other inclusion criteria in Mashhad in the spring of 2018.	absence of more than 2 sessions, non-attendance, counselling and psychotherapy, sessions, psychotic disorders, and addiction.	14(0)	14(0)	UD	UD	UD	UD	UD	UD	routine medical management	8 weeks	1) baseline 2) 8 wks.	Depression Anxiety Stress Scales (DASS-21)	MBCT had a significant effect (p< 0.0001) on the perception of the disease and the psychological syndrome in the experimental group compared to the control group.
Davis_2015	Davis et al, 2015 [12]	Participants were from the Phoenix, AZ metropolitan area.	diagnosis of Lupus Erythematosus, receiving a cyclical estrogen-replacement therapy	47(0)	44(0)	UD	UD	UD	UD	UD	Mindfulness	Arthritis Education	30 days	1) baseline 2) post-intervention	Coping Strategies Questionnaire (CSQ), Arthritis Self-Efficacy Scale (ASES), Fatigue, Morning stiffness, Positive and Negative Affect Scale - Expanded Form (PANAS-X)	The M group reported higher overall levels of pain control than did the CBT-P (p <0 .01) and E groups (p < 0.02). CBT-P group showed improvement in levels of catastrophizing (p <0 .0003), in disability (p<.0001), and fatigue (p< .0002). The E group showed improvement in levels of perceived pain control (p <0.02) and disability (p < 0.0001), but
				52(0)	44(0)	UD	UD	UD	UD	UD	CBT					

																worsening in levels of catastrophizing (p < 0.02).
DeBrouwer_2011b	DeBrouwer et al, 2011 [13]	RA were recruited from the Department of Rheumatology at the Radboud University Nijmegen Medical Centre and the St Maartenskliniek in Nijmegen, the Netherlands.	severe physical comorbidity (e.g., major cardiac problems, psoriasis, malignancies, severe respiratory or renal insufficiency, hepatitis B, HIV, and insulin-dependent diabetes mellitus); severe psychiatric disturbances that might interfere with the study protocol; pregnancy; illiteracy; use of antidepressants, anxiolytics, or antipsychotics; and psycho-logical treatment.	40(6)	34(9)	UD	UD	57.26(11.8)	60.76(9.2)	12-15 year	Stress management training	routine medical management	2 weeks	1) baseline 2) post intervention 3) 9 wk	Impact of Rheumatic diseases on General Health (IRGL), Disease Activity Score (DAS28)	At the follow-up assessment, the tension was significantly lower in patients in the intervention group than in patients in the control group (p = 0.02) .There was a significantly diminished cortisol response (p = 0.03) and a trend towards a lower total cortisol output (AUCg) in the intervention group compared with the control group (p = 0.06). The autonomic response was similar in the two groups (p = 0.59)

Evers_2002	Evers et al, 2002 [15]	Patients were randomly selected from patient medical records of three rheumatology outpatient clinics in the Netherlands.	any other psychological group or individual treatment	30(0)	29(0)	70	72	53.9 (10.3)	53.5 (12.6)	3 years	CBT	routine medical management	6 months	1) baseline 2) 6 months 3) follow up	Disease Activity Score (DAS28), Impact of Rheumatic diseases on General Health (IRGL), Checklist Individual Strength (CIS), Beck Depression Inventory (BDI), Illness Cognition Questionnaire, Utrechtse Coping List (UCL), Pain Coping Inventory (PCI)	Patients in the CBT condition used significantly more active coping strategies when dealing with stress at post-treatment (P< 0,01), but not at follow-up assessment (P=0,16). Helplessness significantly decreased in the CBT condition at post-treatment and follow-up assessment (P<0,01 and P<0,05). There was no change in control condition. Compliance with RA medication significantly increased in the CBT condition at follow-up assessment (P<0:05), but not at post-treatment (P=0:57), while compliance tended to decrease in the control condition at post-treatment and follow-up assessment

(P=0,06;P=
0,08)

Ferwerda_2017	Ferwerda et al, 2017 [16]	Patients were from Radboud University Medical Centre and from 3 non-academic hospitals in Netherland.	pregnancy, insufficient command of the Dutch language, severe physical or psychiatric comorbidity, current treatment by a CBT therapist, no access to a computer and internet	46(11)	59(2)	61	66	55.45 (10.69)	57.14 (9.36)	4 years	Internet-based CBT	routine medical management	9 and 65 weeks	1) baseline 2) post intervention 3) 3 months. 4) 6 months 5) 9 months 6) 12 months.	Beck Depression Inventory (BDI), Impact of Rheumatic diseases on General Health (IRGL), Psychological functioning, Checklist Individual Strength (CIS), Physical functioning, RAND-36 Health Status Inventory (RAND-36), Impact on daily life, Rheumatoid Arthritis Disease Activity Index (RADAI), Compliance	Patients who received the internet-based intervention reported a larger improvement in psychological functioning compared with the control group, indicating less depressed mood (P <0.001), negative mood (P < 0.01), and anxiety (P<0.001) during the 1-year follow-up period. The intervention group reporting less fatigue than the control group (P< 0.06), whereas no effect was found on pain. No effects were found for the impact of RA on daily life, except for the intervention group experiencing fewer role limitations due to emotional problems (P<0.001).

Fogarty_2019	Fogarty et al, 2019 [17]	Patients were from two public hospitals in Auckland, New Zealand.	not according to the 1987 American College of Rheumatology classification criteria	21(0)	21(0)	91	86	52 (12)	55 (13)	10 years	Mindfulness-based stress reduction (MBSR	routine medical management	8 weeks	1) baseline 2) 2 months 3) 4 months 4) 6 months.	Disease Activity Score (DAS28), Hospital and Anxiety Depression Scale (HADS),	In the MBSR group, greater reduction in DAS28-CRP scores was observed compared with the control group (P=0.01) The MBSR group also showed greater improvements in duration of morning stiffness (P=0.03) and pain scores (P=0.04). These effects were evident post-intervention and at both follow-up time points.

Giraudet-Le Quintrec_2007	Giraudet-Le Quintrec et al, 2007 [18]	Patients were from same medical centre.	current juvenile chronic arthritis, Steinbrocker class IV, pregnancy, presence of RA flare, or patient not able to understand the information	100(0)	100(0)	86.41	85.44	55.32 (11.80)	54.31 (14.37)	11-14 years	Intensive education program	routine medical management	8 week	1) baseline 2) 1 yr.	Nocturnal awakenings, Morning stiffness, Disease Activity Score (DAS28), Health Assessment Questionnaire (HAQ), Hospital and Anxiety Depression Scale (HADS), Arthritis Helplessness Index (AHI), Arthritis Impact Measurement Scales 2 Short Form (AIMS2-SF), Functional Assessment of Chronic Illness Therapy (FACIT), Baecke Physical Activity Questionnaire (BPAQ), Knowledge Questionnaire (KQ), Patient's satisfaction	After 1 year, no statistically significant difference was observed between the 2 groups in change in HAQ score (p = 0.79). Statistically significant differences were found in 3 domains: patient coping (p = 0.03), knowledge (p < 0.0001), and satisfaction (p = 0.02), all of which were better for the group attending the education sessions.

Hammond_2004b	Hammond et al 2004 [19]	Participants were followed up from an earlier study.	under 18 and over 65 years, not diagnosed with RA by a rheumatology consultant within the last five years;	65(0)	62(0)	UD	UD	52	51	1-2 years	Joint-protection programme	routine medical management	Two 8-hour interventions	1) baseline 2) 4 yr.	Visits to doctor last 6/12	At four years, the joint protection group continued to have significantly better: joint protection adherence (p=0.001); early morning stiffness (p=0.01); AIMS2 activities of daily living (ADL) scores (p=0.04) compared with the standard group. The joint protection group also had significantly fewer hand deformities: metacarpophalangeal (MCP) (p =0.02) and wrist joints (p =0.04).

Hewlett_2019b	Hewlett et al, 2019 [20]	Patients were approached in clinic or by mailshots to departmental databases.	recent changes to glucocorticoids (6 weeks) or major RA medication (16 weeks) or insufficient English to participate in group discussion	156(0)	152(0)	29221	29007	UD	UD	10 year	Reducing arthritis Fatigue with CBT	routine medical management	26 weeks	1) baseline 2) 26 wks.	Bristol Rheumatoid Arthritis Fatigue - Numerical Rating Scale (BRAF-NRS), Bristol Rheumatoid Arthritis Fatigue - Multidimensional Questionnaire (BRAF-MDQ), Numerical Rating Scale (NRS), Modified Health Assessment Questionnaire (M-HAQ), Arthritis Impact Measurement Scale (AIMS), Simplified Patient-Devised Disease Activity (SPDAS2), Hospital and Anxiety Depression Scale (HADS), Valued Life Activities (VLA), Arthritis Helplessness Index (AHI), Rheumatoid Arthritis	At 26 weeks, the adjusted difference between arms for fatigue impact change favoured RAFT (BRAF-NRS Effect −0.59, 95%CI –1.11 to -0.06), BRAF Multidimensional Questionnaire (MDQ) Total −3.42 (95% CI –6.44 to -0.39), Living with Fatigue −1.19 (95% CI –2.17 to -0.21), Emotional Fatigue −0.91 (95% CI –1.58 to -0.23); RA Self-Efficacy (RASE, +3.05, 95%CI 0.43 to 5.66) (14 secondary outcomes unchanged). Effects persisted at 2 years: BRAF-NRS Effect −0.49 (95% CI −0.83 to -0.14), BRAF MDQ Total −2.98 (95% CI −5.39 to -0.57), Living with Fatigue −0.93 (95% CI −1.75

Self-Efficacy Scale (RASE)	to -0.10), Emotional Fatigue −0.90 (95% CI −1.44, to -0.37); BRAF-NRS Coping +0.42 (95% CI 0.08 to 0.77) (relevance of fatigue impact improvement uncertain).

Hewlett_2011	Hewlett et al, 2011 [21]	Participants were from two teaching hospitals in Bristol, UK.	change in disease-modifying drugs or biological agents within the preceding 24 weeks	40(0)	43(0)	27485	71	58.25(12)	61.1(10)	14-16 year	CBT and self-management:	routine medical management	18 weeks	1) baseline 2) 18wks	Multi-Dimensional Assessment of Fatigue (MAF), Visual Analogue Scale (VAS), Health Assessment Questionnaire (HAQ-DI), Personal Impact HAQ (PIHAQ), Rheumatoid Arthritis Quality-of-Life (RAQoL), Hospital and Anxiety Depression Scale (HADS), Arthritis Helplessness Index (AHI), Rheumatoid Arthritis Self-Efficacy Scale (RASE	At 18 weeks CBT participants reported better scores than control participants for fatigue impact: MAF (p=0.008); VAS (p<0.001).

John_2013	John et al, 2013 [22]	Participants were recruited from a secondary care setting from a single rheumatology centre.	<18 years, not speaking English	52(0)	58(0)	71.15	74.14	62.19 (10.59)	60.81 (10.67)	11-14 year	Cognitive behavioural education intervention	routine medical management	8 weeks	1) baseline 2) 2 months 3) 6 months	Heart Disease Fact Questionnaire Rheumatoid Arthritis (HDFQ-RA), Attitude to behaviour change, Perceived behavioural control over behaviour change, Behavioural intention towards behaviour change, Body Mass Index (BMI)	At 6 months, those in the intervention group had significantly higher knowledge scores (P < 0.001); improved behavioural intentions to increase exercise (P < 0.001), eat a low-fat diet (P = 0.01) and lose weight (P = 0.06); and lower mean diastolic blood pressure by 3.7 mmHg, whereas the control group's mean diastolic blood pressure increased by 0.8 mmHg.

Keefe_2008	Keefe et al, 2008 [23]	Patients were recruited from clinics affiliated with the Ohio University College of Osteopathic Medicine or Duke University Medical School.	other organic disease that would significantly affect function or rheumatic disorders other than RA. Patients with severe personality disorders (e.g., borderline personality disorder), substance abuse problems, or who were involved in current psychiatric treatment	17(2)	16(4)	UD	UD	UD	UD	14 years	Private Emotional disclosure	routine medical management	2 months	1) baseline 2) 2 months 3) 5 months 4) 15 months	Arthritis Impact Measurement Scale (AIMS), Positive and Negative Affect Scale (PANAS), Daily Stress Inventory (DSI), Pain behaviours	There were some benefits in terms of a reduction in pain behaviour with private disclosure vs. clinician-assisted disclosure at the 2-month follow-up, but no other significant between group differences.
				24(2)	16(4)	UD	UD	UD	UD		Clinician assisted Emotional disclosure	routine medical management				
				29(4)	16(4)	UD	UD	UD	UD		Arthritis education	routine medical management				

Kilic_2021	Kilic et al, 2021 [24]	Patients, who applied to the rheumatology outpatient clinic of a university hospital between January and May 2018.	diagnosed with RA for less than 6 months,<18 years old, using continuous sleep medications, any physical problems that would prevent doing the exercises, any cognitive disorder	35(0)	37(0)	28126	28581	46.3 (13.4)	56.6(1 1.2)	8-10 years	Progressive muscle relaxation	routine medical management	6 weeks	1) baseline 2) 6 wks.	Pittsburgh Sleep Quality Index (PSQI), Fatigue Severity Scale (FSS)	A statistical difference was found between the two groups in terms of the Pittsburgh Sleep Quality Index's and Fatigue Severity Scale's mean scores.

Kirwan_2005	Kirwan et al, 2005 [25]	Patients who had previously attended an outpatient education programme were excluded from the study.	UD	30(0)	28(0)	23071	75.0	20546	20821	13-16 years	Patient education	routine medical management	8 weeks	1) baseline 2) 4 weeks 3) 8 weeks 4) 12 weeks 5) 24 weeks 6) 36 weeks	Visual Analogue Scale (VAS), Arthritis Self-Efficacy Scale (ASES)	In those randomized to be offered education, knowledge of RA and its treatment increased by 18% compared to 9% in controls (p = 0.058). Self-efficacy for pain improved between weeks 0 and 4 by 10.3% (p = 0.015) in those offered education, and by 14.1% in those who were offered and accepted education (p = 0.001) but the difference from controls was not maintained after four weeks.

Kraaimaat_1995	Kraaimaat et al, 1995 [27]	Participants were from 4 hospitals in the center of the Netherlands	difficulty ambulating due to aging or medical problems, Class IV RA patients with the most advanced disease	24(0)	19(0)	UD	UD	UD	UD	>1 year	CBT		routine medical management	10 weeks	1) baseline 2) 10 wks 3) 6 months	Impact of Rheumatic diseases on General Health (IRGL),	CBT resulted in minor changes in pain coping behaviour at posttreatment, and showed an increase of knowledge of RA. No therapeutic effects about health status were demonstrated.

Lumley_2011	Lumley et al, 2011 [29]	Adults who met American College of Rheumatology criteria for nonjuvenile RA from one of several urban or suburban rheumatology clinics.	a lack of RA-related pain and disability, physician suspected or diagnosed cognitive impairment (dementia or psychosis), non-English speaking, the presence of another autoimmune rheumatic disease or other major medical condition for which they were receiving treatment, being physically unable to write or walk, participation in another clinical trial, or planning to leave the area within 6 months.	43(0)	45(0)	80	UD	UD	UD	11 years	Writing emotional disclosure	positive or neutral events (combined)	1 month	1) baseline 2) 1month 3) 3 months 4) 6 months	McGill Pain Questionnaire - Short Form (MPQ-SF), Arthritis Impact Measurement Scales - 2 (AIMS2), Pain behaviours, Grip strength, Walking speed, Swollen joint count, Physician's global rating of disease activity, Erythrocyte sedimentation rate (ESR)	The written disclosure had minimal effects in pain compared with combined control. Spoken disclosure led to faster walking speed at 3 months, and reduced pain, swollen joints, and physician-rated disease activity at 6 months. Written disclosure improved affective pain and walking speed. Spoken disclosure showed only a marginal benefit on sensory pain.

48(0)	45(0)	84,4	UD	UD	UD	11 years	Speaking emotional disclosure	positive or neutral events (combined)	1 month	1) baseline 2) 1 month 3) 3 months 4) 6 months
43(0)	24(0)	79	UD	UD	UD	11 years	Writing emotional disclosure	Positive Control - write about Positive emotional events look at Positive aspects avoid dwelling on negative features	1 month	1) baseline 2) 1month 3) 3 months 4) 6 months
43(0)	21(0)	31229	UD	UD	UD	11 years	Speaking emotional disclosure	Positive Control - write about Positive emotional events look at Positive aspects avoid dwelling on negative features	1 month	1) baseline 2) 1 month 3) 3 months 4) 6 month
43(0)	21(0)	31229	UD	UD	UD	11 years	Speaking emotional disclosure	Neutral Control	1 month	1) baseline 2) 1month 3) 3 months 4) 6 month

				48(0)	21(0)	81	UD	UD	UD	11 years	Writing emotional disclosure	Neutral Control	1 month	1) baseline 2) 1 month 3) 3 months 4) 6 months		
Multon_2001	Multon et al, 2001 [32]	All study participants were recruited from a midwestern Department of Veterans Affairs hospital, a university medical center, and a private rheumatology practice.	a history of organic brain disorder, presence of a psychotic disorder, presence of an uncontrolled medical disorder, presence of a major communication disorder, illiteracy	44(0)	44(0)	UD	UD	UD	UD	12 years	Stress management with CBT	Neutral Control	10 weeks	1) baseline 2) 10 weeks 3) 3-month 4) 15-month	Pain behaviours (McGill Pain Questionnaire), Arthritis Impact Measurement Scales (AIMS 5)	The 3 groups did not differ significantly in the change in pain behaviour at any of the assessment periods. However, persons with RA who had less disease activity tended to exhibit positive changes in pain behaviour over time.
				42(0)	44(0)	UD	UD	UD	UD	12 years	Patient education	Neutral Control	11 weeks	1) baseline 2) 10 wks 3) 3-month 4) 15-month		

Pradhan_2007	Pradhan et al, 2007 [35]	Patients were recruited through advertisements in Baltimore news-papers, presentations to rheumatologists, presentations at community health fairs, and informational flyers widely distributed through the Maryland Chapter of the Arthritis Foundation.	major psychiatric illness, active alcohol or drug dependency, diagnosis of fibromyalgia, inability to attend study sessions, concurrent participation in another clinical trial, scheduled major surgery	28(3)	32(0)	84	91	56(9)	53 (11)	6-11 years	Mindfulness-based stress reduction (MBSR)	routine medical management	8 weeks	1) baseline 2) 2 months 3) 6 months	Symptom Checklist-90-Revised (SCL-90-R), Psychological Well-Being Scales, Mindfulness Attention Awareness Scale (MAAS), Disease Activity Score (DAS28)	There was a 35% reduction in psychological distress among those treated. The intervention had no impact on RA disease activity.
Radojevic_1992	Radojevic et al, 1992 [36]	Participants were recruited from a major university medical centre and from private rheumatologists in the San Diego area.	difficulty ambulating due to aging or medical problems, Class IV RA patients with the most advanced disease	15(0)	15(0)	UD	UD	UD	UD	12 years	Behaviour Therapy with Family Support	routine medical management	6 weeks	1) baseline 2) 6 wk 3) 2month	Arthritis Impact Measurement Scale (AIMS), Centre for Epidemiological Studies - Depression Scale (CES-D), Pain	The behavioural interventions demonstrated significantly greater improvement in joint exam pain at follow-up and reduced

14(0)	15(0)	UD	UD	UD	UD		Behavior Therapy	routine medical management		Management Inventory (PMI), Joint Exam	swelling severity and number of swollen joints at posttreatment and follow-up when contrasted with the two control conditions. The behavioural intervention with family support was superior to all other conditions combined on swelling measures at posttreatment but did not differ from the behaviour therapy without family group at follow up.
15(0)	15(0)	UD	UD	UD	UD		Education and Family support	routine medical management			

Riemsma_2003 b	Riemsma et al, 2003 [37]	Participants were recruited from the outpatient clinics of all 7 rheumatologists from 2 hospitals in the province of Twente, the Netherlands.	residence in a nursing home, younger than 20 years or older than 70 years	61(10)	73(3)	58	62	57.2 (10.3)	57.0 (8.3)	11.7 (9.8)	group education with participation of their significant other	routine medical management	5 week	1) baseline 2) 2 month 3) 6 month 4) 12 months	Arthritis Self-Efficacy Scale (ASES), Endurance - Physical - Relaxation exercises, Self-management, Coping with Rheumatoid Stressors Questionnaire (CORS), Dutch version, Social Support Scale, Arthritis Impact Measurement Scales - 2 (Dutch-AIMS2), Visual Analogue Scale (VAS)	Self-efficacy scores for coping with other symptoms were significantly higher for patients participating in the group education without a partner and significantly lower for patients participating in the group education with a partner. Fatigue increased in patients participating in the group education with a significant other and decreased in patients participating in the group education without a significant other.

58(13)	73(3)	66	62	55.1 (10.3)	57.0 (8.3)	11.7 (9.8)	group education for patients only	routine medical management

Shadick_2013	Shadick et al, 2013 [39]	Participants were recruited from the Brigham and Women's Hospital Arthritis Center in Boston.	if they were in remission according to their rheumatologist, or currently taking part c in an arthritis self-management program	38(0)	40(0)	33664	31898	57.8 (13.8)	58.5 (12.0)	14-19 years	Internal Family Systems-based Psychotherapeutic Intervention	Education group	9 month	1) baseline 2) 3 month 3) 6 month 4) 21 months	Disease Activity Score-28-C-reactive Protein 4 (DAS-CRP4), Rheumatoid Arthritis Disease Activity Index (RADAI), Beck Depression Inventory (BDI), State Trait Anxiety Index (STAI), Self-Compassion Scale (SCS), Arthritis Self-Efficacy Scale (ASES), Short Form-12 (SF-12), Visual Analogue Scale (VAS), Multidimensional Health Assessment Questionnaire (M-HAQ)	Posttreatment improvements favouring the IFS group occurred in overall pain (p = 0.04) and physical function (p = 0.04]. Posttreatment improvements were sustained 1 year later in self-assessed joint pain (p=0.04], self-compassion (p = 0.01) and depressive symptoms (p= 0.01). There were no sustained improvements in anxiety, self-efficacy, or disease activity.

Sharpe_2001	Sharpe et al, 2012 [40]	rheumatology clinics at three hospitals in or near London	known history of mental illness or alcohol or drug abuse	23(0)	22(0)	25324	26481	54.14 (14.29)	56.86 (12.75)	12.63 (8.22) months	Cognitive-behavioural therapy (CBT)	routine medical management	8 weeks	1) baseline 2) post 3) 6 months	Hospital and Anxiety Depression Scale (HADS), Coping Strategies Questionnaire (CSQ), Self-monitored level of subjective pain, Health Assessment Questionnaire (HAQ-DI), Ritchie Articular Index (RAI), Erythrocyte sedimentation rate (ESR), C-reactive protein (CRP),	Significant differences were found between the groups at both post-treatment and 6-month follow-up in depressive symptoms. While the CBT group showed a reduction in depressive symptoms, the same symptoms increased in the Standard group. At outcome but not follow-up, the CBT group also showed reduction in C-reactive protein levels.

Sharpe_2003	Sharpe et al, 2001 [41]	rheumatology clinics at three hospitals in or near London	history of psychotic illness, current alcohol or drug abuse or poor English language skills, insufficient to complete the assessment or treatment	23(4)	22(4)	UD	UD	UD	UD	12.6 (8.2) months	Cognitive-behavioral therapy (CBT)	routine medical management	2 weeks	1) baseline 2) 6 months 3) 18 months	Hospital and Anxiety Depression Scale (HADS), Coping Strategies Questionnare (CSQ), Self-monitored level of subjective pain, Health Assessment Questionnaire (HAQ-DI), Ritchie Articular Index (RAI), Erythrocyte sedimentation rate (ESR), C-reactive protein (CRP)	Significant differences were found between the groups in depressive symptoms. The intervention group-maintained improvements in joint function, although those in routine care made similar improvements over the ensuing 18 months. At follow-up, group differences emerged for disability and anxiety.

Sharpe_2012	Sharpe et al, 2003 [42]	Participants were recruited from consecutive patients at two teaching hospitals and through volunteers from the Arthritis Foundation of NSW newsletter.	patients scheduled for surgery or medication review, a history of psychotic illness, current alcohol or drug abuse, or insufficient English to complete assessment or treatment.	24(2)	25(0)	UD	UD	57.9 (12.9)	54.2 (11.0)	13.63 (14.9) years	Behavior therapy (BT)	wait-list control (WLC)	8 weeks	1) baseline 2) post 3) 6 months	Ritchie Articular Index (RAI), C-reactive protein (CRP), Erythrocyte sedimentation rate (ESR), Hospital and Anxiety Depression Scale (HADS), Health Assessment Questionnaire (HAQ-DI)	Participants who received cognitive components had greater improvements in tender joint counts and C-reactive protein at post-treatment. Those receiving either BT or CT alone improved more on anxiety than CBT or WLC. At 6 months, the three active treatment groups could only be distinguished on tender joints, which favored CT and CBT.
				24(2)	25(0)			55.2 (13.3)	54.2 (11.0)		Cognitive therapy (CT)					
				25(2)	25(0)			57.7 (15.4)	54.2 (11.0)		Cognitive-behavioral therapy (CBT)					
Shearn_1985	Shearn et al, 1985 [43]	Oakland medical center and at neighboring Kaiser Permanente facilities. Contacted by telephone and invited to participate.	UD	19(0)	28(0)	77	77	56.4 (11.73)	55 (3.29)	10.19 years	Stress management training	no intervention	10 weeks	1) baseline 2) post	Ritchie Articular Index (RAI), Morning stiffness, Visual Analogue Scale (VAS), Disability scale by Fries, Grip	Patlents In the Intervention groups showed greater improvement in joint tenderness than did the control

										strength, Walking speed, Erythrocyte sedimentation rate (ESR), Center for Epidemiological Studies - Depression Scale (CES-D)	patients ($p < 0.05$)
23(0)	28(0)	72	77	57.9 (2.5)	55 (3.29)		Mutual support group				

Shigaki_2013	Shigaki et al, 2008 [44]	nationwide convenience sample of adults with RA was recruited using a predominantly passive online recruitment approach	previous exposure to self-management interventions, uncontrolled psychiatric diagnoses, uncontrolled medical comorbidities (e.g., active cancer)	44(-)	49(-)	93	92	50.3 (11.6)	49.3 (12.3)	7.94 years	RAHelp (Patient education)	wait-list, offered their choice of treatment following the study	10 weeks	1) baseline 2) post 3) 9 month.	Arthritis Impact Measurement Scales - 2 (AIMS2), Arthritis Self-Efficacy Scale (ASES), Center for Epidemiological Studies - Depression Scale (CES-D), Rapid Assessment of Disease Activity in Rheumatology (RADAR), Quality of Life Scale (QLS), Social Provisions Scale (SPS), Los Angeles Loneliness Scale, version 3 (LS-3)	Group differences with were found immediately postintervention for self-efficacy (P = 0.00001) and quality of life (P = 0.003), respectively. At 9 months postintervention, differences in self-efficacy (P = 0.00001) and quality of life (P = 0.004) remained robust.

vanMiddendorp_2009	vanMiddendorp et al, 2009 [48]	Participants were recruited by rheumatologists and rheumatology nurses of the rheumatology divisions of eight hospitals in the Utrecht area, The Netherlands, participating in the Utrecht Rheumatoid Arthritis Cohort study group.	UD	40(0)	28(0)	60	71	58.7 (11.5)	59.6 (11.4)	12.9 years	Oral emotional disclosure	time management	4 weeks	1) baseline 2) 1 wk 3) 3 months	Impact of Rheumatic diseases on General Health (IRGL)	Cortisol (p = 0.01) and the serum level of the pro-inflammatory cytokine IFN-gamma (p = 0.05) were differentially affected by the two conditions

Wetherell_2005	Wetherell et al, 2005 [49]	Potential patients were first identified by a consultant rheumatologist and then approached by a researcher who was blinded to subsequent group allocation.	undergoing any form of psychotherapy, diagnosis of dementia, any other rheumatological or other major illness	19(0)	15(0)	79	87	62.7 (13.6)	58.6 (14.7)	14.94 years	Written or oral emotional disclosure	Write about daily events	4 days	1) baseline 2) 1 wk 3) 6 wks 4) 10 wks	Disease Activity Score (DAS28), Patient Global Visual Analouge Scale (PG-VAS), Erythrocyte sedimentation rate (ESR), C-reactive protein (CRP), The Short Form of Profile of Mood States (POMS-SF)	The disclosure group demonstrated increases in negative mood and objective markers of disease activity at 1 week postintervention. However, there were significant trends for the disclosure group to demonstrate minor improvements in mood and stability in disease activity, compared with the control group.

Zautra_2008	Zautra et al, 2008 [54]	Participants were recruited from the Phoenix, AZ region via solicitations at health fairs, to Arthritis Foundation members, and at local physicians' offices as well as from rheumatologist referrals at the Carl T. Hayden Veterans Affairs (VA) Medical Center in Phoenix	taking any cyclical estrogen replacement therapies, Lupus diagnosis	6(0)	14(0)	83,3	78,6	46.17 (12.70)	51.43 (13.89)	12.67 years	Mindfulness meditation and emotion regulation therapy	education	30 days	1) baseline 2) post 3) 6 months	Numerical Rating Scale (NRS), Positive and Negative Affect Scale (PANAS), Checklist based on DSM-IV criteria, Coping efficacy question, Coping Strategies Questionnaire (CSQ), Numerical Rating Scale (NRS), Disease Activity Score (DAS28), Interleukin 6	Participants receiving CBT showed the greatest Pre to Post improvement in self-reported pain control and reductions in the IL-6; both CBT and Mindfulness groups showed more improvement in coping efficacy than did the Education group. The relative value of the treatments varied as a function of depression history. RA patients with recurrent depression benefited most from Mindfulness across several measures, including negative and positive affect and physicians' ratings of joint tenderness, indicating that the emotion regulation aspects of that treatment were most beneficial

to those with
chronic
depressive
features.

				17(0)	14(0)	88,2	78,6	51 (10.74)	51.43 (13.89)		Cognitive-behavioral therapy (CBT)					
				41(0)	30(0)	53,7	76,7	57.29 (15.29)	52.43 (12.96)		Mindfulness meditation and emotion regulation therapy					
				35(0)	30(0)	60	76,7	56.11 (13.49)	52.43 (12.96)		Cognitive-behavioral therapy (CBT)					
Zhao_2019	Zhao et al, 2019 [55]	The participants were recruited from a university-affiliated and governmental hospital with 4,300 beds in Sichuan Province, China (West China Hospital, Sichuan University, Chengdu).	no phone equipment, hearing or language barriers and severe cognitive impairment.	43(3)	39(7)	26908	25355	56.93 (11.14)	54.15 (10.06)	4 years	Health education by telephone follow-up (HEFT)	no intervention	12 weeks	1) baseline 2) post 3) 24 wks	Disease Activity Score (DAS28)	The RASE score of the intervention group was higher than that of the control group ($p < .05$) at the 12th week and the 24th week.

Zuidema_2019a	Zuidema et al, 2019 [56]	Between December 2014 and June 2015, patients with a diagnosis of RA aged 18 years or older were invited by a letter to participate in this study, in collaboration with rheumatologists, until the required number of 190 patients was reached.	receiving psychiatric or psychological treatment	57(21)	75(4)	65	66	61.0 (11.3)	62.9 (10.2)	UD	Web-based self-management enhancing program	routine medical management	12 months	1) baseline 2) 6 months 3) 12 months	Patient Activation Measurement (PAM-13), Self-Management Ability Scale (SMAS-S), Rheumatoid Arthritis Self-Efficacy (RASE), Perceived Efficacy in Patient-Physician Interaction (PEPPI-5), RAND-36 Health Status Inventory (RAND-36), Numerical Rating Scale (NRS), Modified Pain Coping Inventory for Fatigue (MPCI-F)	No positive effects were found regarding the outcome measurements.

Zwikker_2014	Zwikker et al, 2014 [57]	Patient inclusion took place between September 2009 and February 2011 at the Sint Maartenskliniek (SMK Nijmegen, the Netherlands), a clinic specialized in rheumatology, rehabilitation and orthopedics.	severe mental or physical constraints or illiteracy in the Dutch language	55(4)	60(0)	24289	26115	60.4 (12.1)	59.3 (11.3)	14.8 years	Motivational interview (MI)	received brochures at home about the disease-modifying anti-rheumatic drugs (DMARDS)	1 week	1) baseline 2) 1 wk 3) 6 months 4) 1 yr	Beliefs about Medicines Questionnaire (BMQ)	At 12 months' follow-up: participants in the intervention arm had less strong necessity beliefs about medication than participants in the control arm

Alleva_2018	Alleva et al, 2018 [1]	Participants were recruited via the website, mailing list, and social media of the National Rheumatoid Arthritis Society, or other relevant media such as private Facebook groups for individuals with rheumatoid arthritis, and other related charities.	UD	40(9)	34(1)	100	100	UD	UD	11.32 (10.92) years	Expand Your Horizon intervention	wait-list control (WLC)	4 days	1) baseline 2) post 3) 1 wk 4) 1 month	Functionality Appreciation Scale (FAS), Body Appreciation Scale-2 (BAS-2), Multidimensional Body–Self Relations Questionnaire - Body Areas (BASS), Body Experience Questionnaire (BEQ), Health Assessment Questionnaire (HAQ-DI), Pain Disability Index (PDI), Patient-Reported Outcomes Measurement Information System (PROMIS)	Relative to control, participants in the intervention experienced improvements in various aspects of body image (functionality appreciation, body appreciation, body satisfaction, body–self alienation) and decreases in depression, with effects persisting at 1-week and 1-month follow-up.

ElMiedany_2012	ElMiedany et al, 2012 [14]	UD	UD	74(0)	73(0)	26085	26908	53.2 (9.6)	52.8 (9.5)	11.25 years	Joint-fitness program	routine medical management	18 months	1) baseline 2) 18 months	Patient Reported Outcome Measures (PROM), Disease Activity Score (DAS28)	The integration of patient education and PROMs led to a significant greater reduction of disease activity parameters, DAS-28 score, as well as improvement of the patients' adherence to therapy (p<0.01). The improvement of disease activity parameters was associated with the improvement in functional disability and quality of life scores. At the 18-month follow-up, both the self-management and cognitive behavioural therapy intervention demonstrated improvement for disease activity.

Knittle_2013	Knittle et al, 2013 [26]	Patients who had attended the outpatient rheumatology department of either Leiden University Medical Center, HAGA Hospital, or Reinier DeGraaf Gasthuis were potentially eligible for study participation. Randomly selected groups of 250 eligible patients were mailed leaflets describing the study.	5×30 physical activity (PA) recommendation met, physical therapy for RA within the last 6 months received, difficulty ambulating.	38(0)	40(0)	79	55	60.7 (11.9)	64.7 (11.5)	UD	Motivational interview (MI)	group-based patient education session	5 weeks	1) baseline 2) 6 wks 3) 32 wks	Short Questionnaire to Assess Health-Enhancing PA (SQuAsH), Days per week with at least 30 min of physical activity, Treatment Self-Regulation Questionnaire (TSRQ-15), Self-Efficacy to Regulate Excercise Scale, Rheumatoid Arthritis Disease Activity Index (RADAI), Health Assessment Questionnaire (HAQ), Brief Symptom Inventory (BSI), Checklist of Individual Strengths (CIS-20)	Significant treatment effects were found for leisure-time PA (p =0.022), active days/week (p =0.016), self-efficacy (p =0.008) and autonomous motivation (p =0.001). At post-treatment and 6- months follow-up, significantly more treated patients than controls met current PA recommendations. Combining motivation- and action-focused intervention approaches improved PA-related cognitions and led to improved uptake and maintenance of leisure-time PA.

Lumley_2014	Lumley et al, 2014 [28]	The Michigan site recruited largely through community advertisements, flyers in rheumatologists' offices, and letters sent to patients from the local Arthritis Foundation. The North Carolina site recruited directly through its rheumatology clinics, and the initial contact screening, including confirmation of criteria, was conducted by referring rheumatologists and staff at those clinics.	another autoimmune disorder, current life-threatening disease (e.g., cancer), illiteracy or cognitive impairment, participation in a formal behavioral pain management program, experience of a major stressful life change in the prior 6 months, inability to write or walk	67(0)	65(0)	29373	28703	56.0 (10.4)	55.3 (11.9)	13.1 (11.4) years	Written emotional disclosure (WED) + Coping skills training (CST)	control writing	8 weeks	1) baseline 2) 1 month 3) 4 months 4) 12 months	Visual Analouge Scale (VAS), Arthritis Impact Measurement Scales - 2 (AIMS2), McGill Pain Questionnaire (MPQ), Time to walk 50 feet, Inflammatory activity	Compared to control training, CST decreased pain and psychological symptoms through 12 months. The effects of WED were mixed: compared with control writing, WED reduced disease activity and physical disability at 1 month only, but WED had more pain than control writing on one of two measures at 4 and 12 months.

69(0)	65(0)	29618	28703	55.2 (12.3)	55.3 (11.9)	Written emotional disclosure (WED)
63(0)	65(0)	30682	28703	54.0 (13.7)	55.3 (11.9)	Coping skills training (CST)

Scholten_1999	Scholten et al, 1999 [38]	UD	UD	38(0)	30(0)	UD	UD	UD	UD	8.9 (1.2) years	Multidisciplinary Arthritis Training Program	waiting-list control group	9 days	1) baseline 2) 2 wks 3) 6 wks 4) 52 wks	Health Assessment Questionnaire (HAQ-DI), Freiburg Questionnaire of Coping with Illness (FQCI), Beck Depression Inventory (BDI)	A significant and persistent improvement of all investigated parameters was demonstrated in the l-year controlled trial. parameters was demonstrated in the l-year controlled trial. Between the end-point of the l-year study and the 5-year evaluation, this improvement increased even more for functional status and coping with illness, whereas depression returned to baseline values.

Smyth_1999	Smyth et al, 1999 [45]	Volunteers recruited from local communities who had RA	ongoing psychotherapy, psychiatric disorder, symptom report interfering medication usage, more than 10 mg of prednisone daily dosage, inability to write for a duration of 20 minutes	31(1)	17(0)	UD	UD	UD	UD	UD	Emotional disclosure	control writing	3 days	1) baseline 2) 2 wks 3) 8 wks 4) 16 wks	Physician's global rating of disease activity	Rheumatoid arthritis patients in the experimental group showed improvements in overall disease activity (a mean reduction in disease severity from 1.65 to 1.19 [28%] on a scale 0 [asymptomatic] to 4 [very severe] at the 4 month followup; P = .001, whereas control group patients did not change.

Zangi_2012	Zangi et al, 2012 [53]	Recruited from three rheumatology departments in south-eastern Norway between March 2007 and June 2009.	Inability to understand Norwegian	34(2)	34(1)	28338	80.0	53.0 (9.4)	54.9 (8.9)	16.2 (12.7) years	Mindfulness based group intervention (Vitality Training Programme - VTP)	routine medical management	15 weeks	1) baseline 2) post 3) 12 months	General Health Questionnaire (GHQ), Arthritis Self-Efficacy Scale (ASES), Emotional Approach Coping Scale (EAC), Numerical Rating Scale (NRS)	Significant treatment effects in favour of the VTP group were found post-treatment and maintained at 12 months in psychological distress, self-efficacy pain and symptoms, emotional processing, fatigue, self-care ability and overall well-being.

Hadi	Yousefi et al, 2015 [51]	Patients attending the community based Rheumatology clinic	American Rheumatism Association (ARA) class IV (unable to do self care), arthritis other than RA, positive history of mental illness or alcohol or drug abuse, medical condition requiring activity to restrict (e.g. history of more severe heart, lung or cerebrovascular disease), previous participation in a similar intervention program in last 1 year, patient not fit to participate as per the discretion of the rheumatologist	96(4)	93(13)	86	32660	42.6 (13.2)	46.6 (10.9)	14.9 years	Modular program group intervention (MPGI)	routine medical management	8 weeks	1) baseline 2) 20 wks 3) 32 wks 4) 48 wks 5) 60 wks	Visual Analogue Scale (VAS), Medical Outcomes Study Short Form 36 (SF-36)	Significant worsening in the control group compared to improvement in the intervention group, at 2nd, 3rd, 4th and 5th evaluations, the improvement was often seen as early as 12-24 week follow up.

Lumley_2011	Lumley et al, 2011 [240]	Recruited adults who met American College of Rheumatology criteria for non-juvenile RA from one of several urban or suburban rheumatology clinics.	Lack of RA related pain and disability, physician-suspected or diagnosed cognitive impairment (dementia or psychosis), illiteracy or non-English speaking, the presence of another autoimmune rheumatic disease or other major medical condition for which treatment is received, being physically unable to write or walk, participation in another clinical trial, or planning to leave the area within 6 months.	43(0)	45(0)	32234	80	55.4 (11.7)	54.3 (10.0)	11.2 years	Emotional disclosure (writing)	Combined control	1 week	1) baseline 2) 1 month 3) 3 months 4) 6 months	McGill Pain Questionnaire - Short Form (MPQ-SF), Arthritis Impact Measurement Scales - 2 (AIMS2), Pain behaviours, Grip strength, Walking speed, Swollen joint count, Physician's global rating of disease activity, Erythrocyte sedimentation rate (ESR)	Written disclosure had minimal effects compared to combined controls—only pain was reduced at 1 and 6 months. Spoken disclosure led to faster walking speed at 3 months, and reduced pain, swollen joints, and physician-rated disease activity at 6 months. Written disclosure improved affective pain and walking speed; spoken disclosure showed only a marginal benefit on sensory pain.
				48(0)	45(0)	30376	30773	53.1 (11.3)	55.5 (11.9)		Emotional disclosure (speaking)	Combined control				
				43(0)	24(0)	32234	79	55.4 (11.7)	53.1 (10.0)		Emotional disclosure (writing)	Positive control				

				48(0)	24(0)	30376	30376	53.1 (11.3)	58.0 (12.2)		Emotional disclosure (speaking)	Positive control				
				43(0)	21(0)	32234	81	55.4 (11.7)	55.7 (10.0)		Emotional disclosure (writing)	Neutral control				
				48(0)	21(0)	30376	31229	53.1 (11.3)	52.6 (11.1)		Emotional disclosure (speaking)	Neutral control				
Masiero_2007	Masiero et al, 2007 [30]	The patients, recruited via invitationto participate, were hospital outpatients from our hospital's rheumatology department.	(a) Previous participation in educational training (b) Variations in drug therapy at any time during the trial and (c) Rehabilitation treatment or orthopedic surgery during the trial	36(0)	34(0)	29342	30042	54.2 (9.8)	52.2 (11.9)	12.8 (8.8) years	Educational-behavioral joint protection	routine medical management	12 weeks	1) baseline 2) 8 months	Arthritis Impact Measurement Scales-2 (AIMS2), Health Assessment Questionnaire (HAQ), Visual Analogue Scale (VAS)	After a mean time of 8 months, the patients receiving educational training displayed a significant decrease, compared to the CG, in the VAS (p=0.001), HAQ (p=0.000), and physical (p=0.000), symptoms (p=0.049), and social interaction (p=0.045) scores on the AIMS2, but not in other items.

Moghadam_2018	Moghadam et al, 2018 [31]	Among 900 patients with rheumatoid arthritis, referring to the rheumatology clinic of Hafez hospital, Shiraz, southwest Iran, 64 women with confirmed RA from May to July 2013 were enrolled.	Changes in the trend of articular therapy and medications during the study or the last 6 months, need to change the treatment, undergoing joint surgery during the study or 6 months before the intervention, participation in a similar training programs and absence in more than two sessions of the training program.	32(0)	32(0)	100	100	48.06 (10.51)	48.87 (9.24)	UD	Group education program	routine medical management	8 weeks	1) baseline 2) post 3) 3 months	Arthritis Self-Efficacy Scale (ASES)	Mean of self-efficacy scores of the intervention group, immediately and three months after the intervention, significantly enhanced in all dimensions compared with the control group (P<0.001, P<0.001).

Nia_2018	Nia et al, 2018 [33]	Patients with RA referring to a rheumatology clinic in Yasuj City, 2016.	High score of pain (mild pain) based on scale, patient's unwillingness to participate, hearing and vision problems, patient's immigration or death, and having an unpleasant memory of forests and natural sceneries.	25(0)	25(0)	UD	UD	UD	UD	UD	Eye movement desensitization and reprocessing (EMDR)	waiting-list control group	6 days	1) baseline 2) post	Rheumatoid Arthritis Pain Scale (RAPS)	A significant difference was observed in the mean pain score between EMDR and guided imagery groups, and also between each intervention group and the control group (P=0.001).
				25(0)	25(0)	UD	UD	UD	UD		Guided imagery					

Nunez_2006	Nunez et al, 2006 [34]	Patients who agreed to participate in the program and gave informed consent were referred from the outpatient clinic of the Rheumatology department of the Hospital Clinic, a tertiary care center in Barcelona, Spain, to the therapeutic education and functional readaptation (TEFR) Unit of the musculoskeletal clinic institute of the same hospital.	illiteracy; disease onset <16 years of age; other inflammatory joint disease in addition to RA; pain due to infection, metabolic or neoplasic disease; diagnosis of severe psychopathology.	22(0)	21(0)	29	36	51.09 (16.62)	55.40 (16.32)	20.5 months	Education intervention	routine medical management	1 year	1) baseline 2) 18 months	Health Assessment Questionnaire (HAQ-DI), Visual Analogue Scale (VAS), Erythrocyte sedimentation rate (ESR), C-reactive protein (CRP), Visual Analogue Scale (VAS)	At 18 months, patients in the intervention group had less disability (HAQ), pain intensity, number of tender and swollen joints, and patient's and physician's global assessments (p=0.003, 0.031, 0.003, 0.001, 0.014, and 0.004, respectively) compared with baseline, and improvements in disability and number of tender and swollen joints (p=0.024, 0.040, and 0.003, respectively), compared with controls.

Song_2020	Song et al, 2020 [46]	Potential patients were recruited from a department of rheumatology, at a tertiary hospital in Chengdu, Sichuan province, China.	(a) hearing impairment; (b) lack of telephone; (c) severe cognitive or mental disorder; (d) participation in other educational programs.	41(0)	36(0)	26696	25294	57.05 (11.31)	53.22 (10.04)	UD	Telehealth educational intervention	routine medical management	12 weeks	1) baseline 2) 12 wks 3) 24 wks	Disease Activity Score (DAS28), Erythrocyte sedimentation rate (ESR)	The intervention group had significantly higher medication adherence compared with the control group at 12th and 24th week.

Taibanguay_2019	Taibanguay et al, 2019 [47]	Patients who fulfilled the 2010 American College of Rheumatology/European League against Rheumatism criteria for RA were recruited from rheumatology clinic of the Phramongkutklao Hospital from March 2017 to February 2018.	<18 years of age, diagnosed with life-threatening conditions, unable to read Thai, unable to take medication by him/herself, high disease activity (disease activity score-28, [DAS28] .5.1), severe mental disorder.	60(0)	59(0)	85	30317	55.82 (11.25)	57.20 (12.24)	8.01 years	Multi-component intervention group	routine medical management	30 minutes	1) baseline 2) 12 wks	Medication-taking Behavior Questionnaire (MTB), Disease Activity Score (DAS28), EuroQol 5 Dimensions (EQ-5D-5L), EuroQol Visual Analog Scale (EQ-VAS), Visual Analogue Scale (VAS), Brief Illness Perception Questionnaire (B-IPQ)	After 12 weeks, the pill count adherence rate increased significantly from baseline in both study groups. In the multi-component intervention group, adherence rate increased from 92.21±14.05 to 97.59±10.07 (P=0.002) and in the single intervention group, it increased from 88.60±19.66 to 92.42±14.27 (P=0.044).

Yazdani_2017	Yazdani et al, 2017 [50]	The research population included the clients with RA who had referred to Arya Hospital for treatment purposes.	Reluctance to continue participating in the intervention, absence for more than two sessions and inability to perform PMR due to increased pain or other reasons.	31(0)	31(0)	29373	30560	50.3 (9.65)	48 (9.19)	UD	Progressive muscle relaxation	no intervention	8 weeks	1) baseline 2) post	Medical Outcomes Study Short Form 36 (SF-36)	The findings of the study showed that, in the experimental group, the mean score of life quality changed from 37.84 to 54.54 after the intervention (p=0.00001), while, in the control group, it altered from 37.47 to 43.20.There was a significant difference between experimental and control groups regarding the six aspects of life quality including physical function (p=0.041), vitality (p=0.029), social function (p=0.017), mental health (p=0.001), general health (p=0.002), and psychological health (p=0.002).
Yousefi_2022	Yousefi et al, 2022 [52]	Participants included all patients with rheumatoid	Any joint disease other than rheumatoid	19(0)	19(0)	30713	30713	51.26 (5.70)	50.89 (6.84)	UD	Mindfulness Based Stress Reduction (MBSR)	routine medical management	8 weeks	1) baseline 2) post	Chalder Fatigue Scale (CFS), Pittsburgh	At post-treatment, both MBSR and CBT groups

arthritis referred to the rheumatology office in Kashan.	arthritis, drug use if, during the research, the participant for any reason needs a drug other than the prescribed drugs will not be considered despite attending meetings in the study, alcohol consumption, absence from more than one session in MBSR sessions, absence from CBT sessions for more than two sessions.	3) 3 months	Sleep Quality Questionnaire (PSQQ), Tower of London test (TOL), Disease Activity Score (DAS28)	were significantly more effective than the control group on all variables (P < .001) except disease activity (P > .05). In the post-test and Follow-up stages, the mean of chronic fatigue and sleep quality in the intervention groups (MBSR and CBT) was significantly lower than in the control group; also, concerning the executive performance variable, in the post-test stage, the average executive performance in the CBT group was significantly higher than the control group and in the Follow-up stage, the average of both intervention groups was significantly higher than the control group.

19(0)	19(0)	30713	30713	48.73 (7.30)	50.89 (6.84)	Cognitive-behavioural therapy (CBT)	10 weeks
